# Integration of metabolomics and machine learning algorithm for discovery of early diagnostic biomarkers of osteoporosis

**DOI:** 10.1007/s11306-026-02506-5

**Published:** 2026-07-14

**Authors:** Jing Liu, Jialong Wang, Zhijun Bao, Jingkun Jia, Jinru Jia, Lifeng Han, Jinghui Du, Erwei Liu

**Affiliations:** 1 State Key Laboratory of Component-Based Chinese Medicine, Instrumental Analysis & Research Center, Tianjin University of Traditional Chinese Medicine, 10 Poyanghu Road, Jinghai District, Tianjin, 301617 P.R. China; 2 First Teaching Hospital of Tianjin University of Traditional Chinese Medicine, National Clinical Research Center for Chinese Medicine, Tianjin, 300381 P.R. China; 3 Clinical College of Orthopedics, Tianjin Medical University, Tianjin, 300211 P.R. China; 4 SCIEX China, 5 F, 1# building, 24# Yard, Jiuxianqiao Mid Road, Chaoyang District, Beijing, 100015 P.R. China

**Keywords:** Osteoporosis, Metabolomics, Lipidomics, Biomarkers, Machine learning

## Abstract

**Background:**

Osteoporosis (OP) is a prevalent metabolic bone disorder and a major public health concern characterized by reduced bone mass and bone microstructural deterioration. Early identification of osteoporosis and implementation of preventive interventions remain critical for reducing fracture risk and disease burden. Identifying plasma biomarkers reflecting metabolic alterations related to OP may facilitate early detection and risk assessment.

**Methods:**

Untargeted metabolomics and lipidomics profiling based on ultra-performance liquid chromatography-mass spectrometry (UHPLC-MS) was performed in a discovery cohort comprising 75 patients with OP and 140 healthy controls. Differential features were identified using multivariate statistical analysis (PCA, OPLS-DA), and FDR-adjusted univariate analysis. Multivariable logistic regression and LASSO-regularized logistic regression was employed, with age and sex incorporated as mandatory covariates to identify independent lipid predictors. A Random Forest (RF) model was further evaluated in an independent validation cohort consisting of 20 OP patients and 42 healthy controls.

**Result:**

Sixty-one differential metabolites were identified, primarily enriched in lipid metabolism pathways. Further targeted lipidomics identified four diagnostic lipid biomarkers, including LPA(16:0), LPI(16:0), LPI(18:0), and LPI(20:0). Following covariate adjustment for age and sex, key lipid species remained independently associated with OP. The RF-based diagnostic model maintained robust performance in the validation cohort, yielding an AUC of 0.916 (95% CI: 0.842–0.990), with high sensitivity and specificity.

**Conclusions:**

LPA(16:0), LPI(16:0), LPI(18:0), and LPI(20:0) in plasma were negatively correlated with the T value of bone mineral density. These associations persisted after stringent adjustment for age and sex, suggesting that lysophospholipid dysregulation is an independent metabolic hallmark of OP. The multi-metabolites model based on four biomarkers showed promising predictive performance for OP and may provide a potential tool for early risk assessment.

**Supplementary Information:**

The online version contains supplementary material available at https://doi.org/10.1007/s11306-026-02506-5.

## Introduction

Osteoporosis (OP) is a globally prevalent metabolic bone disorder and an escalating public health concern (Aibar-Almazán et al., 2022). Epidemiological studies estimate that 12–23% of men suffer from OP, whereas the incidence among postmenopausal women increases sharply with advancing age (Black & Rosen, 2016; Keen et al., 2025). With rapid population aging, the global burden of OP and its associated complications is expected to rise substantially in the coming decades (Polyzos et al., 2021).

Progressive reduction in bone mineral density (BMD) and deterioration of bone microarchitecture constitute the pathological hallmarks of OP, leading to increased skeletal fragility and susceptibility to fractures, particularly at the vertebral and hip sites (Adejuyigbe et al., 2023). Osteoporotic fractures carry high morbidity and mortality rates-approximately 20% of patients die within one year of a major fracture, and nearly half of survivors experience long-term disability or impaired mobility (Huo et al., 2024). These outcomes impose profound physical, psychological, and socioeconomic burdens, further straining healthcare systems worldwide (Borgström et al., 2020; Pisani et al., 2016).

Currently, the diagnosis of OP primarily relies on dual-energy X-ray absorptiometry (DXA), supplemented by biochemical bone turnover markers such as plasma β-carboxy-terminal telopeptide of type I collagen (β-CTX) and osteocalcin. However, DXA exhibits limited sensitivity in detecting early bone loss, as measurable bone density decline typically requires a reduction of > 30% in bone mass (Lane, 2006). Moreover, BMD reflects only the mineral component of bone without capturing changes in microstructural integrity or biomechanical properties. This leads to the “bone density paradox,” where individuals with normal bone density may still experience fragility fractures (Kutsal & Ergin Ergani, 2021). Bone turnover markers also exhibit limited specificity and are susceptible to demographic, metabolic, and nutritional factors, restricting their value in early diagnosis and individualized risk assessment.

Due to the insidious progression of osteoporosis and its asymptomatic nature before fractures, many patients are diagnosed only at advanced stages, when structural deterioration is largely irreversible and treatment options are limited. This underscores the urgent need for novel biomarkers capable of detecting early metabolic disturbances and improving diagnostic performance before significant bone loss occurs (Wang et al., 2025).

Metabolomics constitutes a vital component of systems biology, offering comprehensive assessment of small-molecule metabolites and capturing dynamic metabolic reprogramming with high sensitivity. Advances in analytical platforms, particularly ultra-high-performance liquid chromatography-mass spectrometry (UHPLC-MS), have enabled high-throughput detection of disease-associated metabolic characters (Wang et al., 2020; Liu et al., 2023). Growing evidence indicates a complex bidirectional regulatory relationship between OP and lipid metabolism, mediated through shared mechanisms such as oxidative stress, chronic low-grade inflammation, and signaling dysregulation (Romero-Márquez et al., 2021; Zheng et al., 2020; Zolfaroli et al., 2021). Elevated triglycerides and low-density lipoprotein cholesterol (LDL-C) are associated with reduced BMD and increased fracture risk, whereas high-density lipoprotein cholesterol (HDL-C) may confer bone protection by promoting osteoblast differentiation and suppressing osteoclast activity (Li et al., 2020; Tang et al., 2021). Conversely, osteogenic hormones like osteocalcin regulate systemic lipid metabolism by enhancing insulin sensitivity and promoting fatty acid oxidation in adipose tissue and liver, suggesting that bone loss may further exacerbate lipid dysregulation through impaired skeletal endocrine function. This interaction underscores the holistic role of lipid metabolism in the pathogenesis of OP. Furthermore, the integration of metabolomics with machine learning algorithm enables robust feature extraction, pattern recognition, and predictive modeling, thereby significantly enhancing the diagnostic accuracy and versatility (Zhang et al., 2025).

This study investigated an independent clinical cohort of osteoporosis (OP) patients using plasma metabolomics and lipidomics based on UHPLC-MS. Differential metabolites were identified through multivariate analyses, including principal component analysis (PCA) and orthogonal partial least squares discriminant analysis (OPLS-DA), followed by KEGG pathway enrichment. A multi-metabolite diagnostic model was constructed using a random forest (RF) algorithm and validated in an independent cohort. Notably, the identified biomarkers remained significantly associated with OP after adjustment for age and sex, indicating disease-specific metabolic alterations. Our findings demonstrate that plasma lipid-related metabolites have strong potential for early OP diagnosis and may contribute to improved risk stratification and preventive strategies.

## Materials and methods

###  Study population and cohort design

Participants in both the discovery and validation cohorts were inpatients recruited from the First Affiliated Hospital of Tianjin University of Traditional Chinese Medicine. The discovery cohort included 215 subjects enrolled between January 2025 and April 2025, comprising a control group (Con, *n* = 140) and an osteoporosis group (OP, *n* = 75). An additional 62 subjects were prospectively recruited between April 2025 and June 2025 using the same inclusion and exclusion criteria, forming the validation cohort (Con, *n* = 42; OP, *n* = 20). Healthy controls were recruited independently from the same study setting. No formal individual matching procedure was performed between the OP and control groups with respect to age or sex.

This study adhered to the ethical principles of the Declaration of Helsinki and was approved by the Ethics Committee of the First Teaching Hospital of Tianjin University of Traditional Chinese Medicine (Approval No. TYLL2025[Z]057). All participants provided written informed consent.

### Inclusion criteria

1. Lumbar or hip T-score ≤ − 2.5 measured by dual-energy X-ray absorptiometry (DXA);
2. Absence of secondary osteoporosis (e.g., glucocorticoid therapy > 3 months);
3. No history of metabolic bone disorders;
4. Complete clinical and laboratory data available.

### Exclusion criteria

1. Presence of hematological, psychiatric, or severe cardiovascular/cerebrovascular diseases;
2. Severe hepatic or renal dysfunction;
3. History of malignant tumors;
4. Prior treatment with anti-osteoporotic medications;
5. Reproductive system disorders (e.g., premature ovarian insufficiency);
6. Contraindications to hormone therapy, including known or suspected sex hormone-related malignancies, suspected or confirmed breast cancer, or unexplained vaginal bleeding;
7. Incomplete clinical data.

Although the exclusion criteria removed participants with major systemic diseases or conditions directly affecting bone metabolism, detailed information on common metabolic comorbidities, chronic inflammatory status, long-term medication use, menopausal status, and hormone therapy was not systematically available for all participants. Therefore, these variables were not incorporated into the exclusion criteria or adjusted for in the present analyses.

### Data collection

#### General clinical information

Demographic and anthropometric data, including gender, age, height, weight, and body mass index (BMI), were recorded. Laboratory and bone-related indicators included alanine aminotransferase (ALT), aspartate aminotransferase (AST), vitamin D (VD), high-density lipoprotein (HDL), low-density lipoprotein (LDL), serum calcium (Ca), serum phosphate (P), lumbar T-score, and hip T-score.

#### Baseline clinical assessment

Clinical indicators were compared between groups to evaluate baseline characteristics and potential confounding factors.

#### Plasma collection

Fasting venous blood was collected from participants diagnosed with OP and immediately centrifuged. Plasma samples were aliquoted and stored at − 80 ℃ within 30 min of collection to ensure metabolic stability.

### Omics analysis

#### Plasma metabolomics analysis based on UHPLC-MS

##### Sample preparation

A total of 100 µL of plasma was mixed with 500 µL of pre-cooled methanol, vortexed for 5 min, and centrifuged at 19,800 *g* for 20 min at 4℃. The supernatant was evaporated under nitrogen, reconstituted in 100 µL of methanol, vortexed, centrifuged again under identical conditions, and the final supernatant was injected for analysis. The quanlity control (QC) samples were mixed with 5 µL of all the samples.

##### Liquid chromatography

Chromatographic separation was performed using an ACQUITY UPLC system equipped with an HSS T_3_ column (2.1 × 100 mm, 1.8 μm) at 35℃. Mobile phases consisted of (A) water with 0.1% formic acid and (B) acetonitrile. The flow rate was 0.3 mL/min with a gradient elution as follows: 0–7 min, 5%–100% B; 7–10 min, 100% B; 10–10.1 min, 100%–5% B; 10.1–13 min, 5% B for column re-equilibration. The injection volume was 2 µL.

##### Mass spectrometry

Metabolomics profiling was conducted on an Orbitrap Exploris 120 mass spectrometer with a heated electrospray ionization (HESI) source. Data were acquired in both positive and negative ion modes using a full MS/dd-MS^2^ strategy. Full MS scans were performed over a mass range of *m/z* 100–1000 at a resolution of 60,000. The top 5 of the most intense ions from the full scan were selected for fragmentation via higher-energy collisional dissociation (HCD) at stepped normalized collision energies of 20%, 40%, and 60%. The MS/MS spectra were acquired at a resolution of 15,000. The source parameters were set as follows: spray voltage, 3.5 kV (positive) and 3.0 kV (negative); sheath gas flow rate, 35 (arbitrary units); auxiliary gas flow rate, 10 (arbitrary units); ion transfer tube temperature, 320℃; and vaporizer temperature, 350 ℃.

#### Plasma lipidomics analysis based on UHPLC-MS

##### Sample preparation

A total of 100 µL of plasma was mixed with 300 µL of pre-cooled isopropanol, vortexed for 5 min, precipitated at − 20℃ for 1 h, and centrifuged at 19,800 *g* for 20 min at 4℃. Then, 100 µL of the supernatant was used directly for lipidomic analysis.

##### Liquid chromatography

Separation was performed using an ACQUITY BEH C_8_ column (2.1 × 100 mm, 1.7 μm). The column temperature was maintained at 50℃. Mobile phase A consisted of water/methanol/acetonitrile (3:1:1, v/v/v) containing 5 mmol/L ammonium acetate; mobile phase B was isopropanol containing 5 mmol/L ammonium acetate. The flow rate was 0.3 mL/min, and the injection volume was 2 µL. The gradient elution program was as follows: 0–0.5 min, 20% B; 0.5–1.5 min, 20%–40% B; 1.5–3 min, 40%–60% B; 3–13 min, 60%–98% B; 13–14 min, 98% B; 14–17 min, 98%–20% B.

##### Mass spectrometry

Lipidomics analysis employed an AB SCIEX QTRAP 6500 + triple quadrupole linear ion trap mass spectrometer coupled with an electrospray ionization (ESI) source. Data acquisition was performed in both positive and negative ion modes under predefined multiple reaction monitoring (MRM) conditions. Ion source parameters were set as follows: ion source temperature 400℃; ion spray voltage 5500 V; gas source 1 (GS1) and gas source 2 (GS2) at 50 psi each; curtain gas pressure 35 psi.

### Quality control and data processing

Quality control (QC) procedures were implemented throughout the untargeted metabolomics and lipidomics workflows to ensure analytical reproducibility and data reliability. A pooled quality control (QC) sample, prepared by combining equal aliquots of all study samples, was injected every 10 study samples throughout the entire analytical sequence to monitor instrument stability and signal drift. Detailed QC feature statistics and CV distributions are provided in the Supplementary Information.

Untargeted metabolomics data were acquired using Xcalibur (Thermo Fisher Scientific) and processed in Compound Discoverer 3.3 for peak detection, alignment, normalization, compound annotation, and statistical filtering. Multivariate analyses, including principal component analysis (PCA) and orthogonal partial least squares discriminant analysis (OPLS-DA), were conducted using SIMCA 14.1. Metabolites were considered potential biomarkers if they met the following criteria: FDR-adjusted *p* < 0.05, fold change (FC) > 1.2 or < 0.833, and variable importance in projection (VIP) ≥ 1. Compound annotation was performed using the HMDB and an in-house database, followed by KEGG pathway enrichment analysis.

Lipidomics data were processed using SCIEX OS for peak integration. Subsequent preprocessing—including missing value imputation, Z-score normalization, and log transformation—was performed using MetaboAnalyst 6.0. Multivariate analyses (PCA and OPLS-DA) were also conducted using SIMCA 14.1. To ensure robust biomarker selection, both univariate and multivariate criteria were applied. Lipid species were considered significant only if they simultaneously met the following thresholds: VIP ≥ 1, FC > 1.2 or < 0.833, and FDR-adjusted *p* < 0.05.

### Metabolite identification and annotation

Metabolite annotation confidence was assigned according to the Metabolomics Standards Initiative (MSI) guidelines. MSI Level 1 metabolite identification was assigned only when authentic reference standards were analyzed under the same experimental conditions using the same LC-MS/MS platform as the study samples. Metabolite identities were confirmed by matching retention time, precursor ion mass, and MS/MS fragmentation spectra between the study samples and the corresponding authentic reference standards. Metabolites without confirmation using authentic reference standards were assigned lower MSI confidence levels according to the available MS/MS spectral matching and annotation evidence.

### Statistical analysis

Statistical analyses were performed using SPSS 26.0 and GraphPad Prism. Data normality was assessed using the Shapiro-Wilk test. Normally distributed variables are presented as mean ± standard deviation (*x* ± *s*) and compared using independent-sample t-tests, while non-normally distributed data are expressed as median (Q1, Q3) and analyzed using the Mann-Whitney U test. Categorical variables are presented as counts (n) and percentages (%) and compared using the chi-square test. A two-sided *p* < 0.05 was considered statistically significant.

To account for potential confounding by demographic variables, covariate-adjusted analyses were performed using generalized linear models, with age and sex included as covariates for comparisons between osteoporosis and control groups. For biomarker evaluation, multivariable logistic regression models were constructed by integrating candidate lipid biomarkers with age and sex. For age- and sex-adjusted analyses, each metabolite or lipid feature was evaluated using a multivariable regression model in which metabolite/lipid abundance was used as the dependent variable, and disease group, age, and sex were included as independent variables. PCA and OPLS-DA were performed using the processed abundance matrix for exploratory visualization, whereas age- and sex-adjusted statistical results were used to support feature selection and biomarker prioritization. Receiver operating characteristic (ROC) curves were generated based on predicted probabilities from the adjusted models to assess diagnostic performance.

For the machine learning analysis, a Random Forest (RF) model was constructed using the randomForest package in R. To ensure model reproducibility and optimal performance, the number of trees (ntree) was set to 500, and the number of variables randomly sampled at each split (mtry) was set to 3. The final model configuration was selected by minimizing the out-of-bag (OOB) classification error rate. The optimized RF model was further evaluated in an independent validation cohort consisting of 20 OP patients and 42 healthy controls. Additional details on data preprocessing, such as missing value imputation and normalization, are provided in the Supplementary Material.

## Results

### Demographic characteristics and analysis of underlying diseases

#### Demographic characteristics analysis

The study summarized the demographic and clinical characteristics of participants in the OP group (*n* = 75) and the Con group (*n* = 140) (Table 1). The gender distribution differed significantly between the two groups (*χ*² = 23.273, *p* < 0.001). The mean age of patients in the OP group (67.85 ± 8.59 years) was substantially higher than that in the Con group (47.30 ± 14.53 years) (*t* = 13.007, *p* < 0.001). Height was significantly lower in the OP group (1.62 ± 0.05 m) than in Con (1.66 ± 0.08 m) (*t* = − 4.892, *p* < 0.001). There were no statistically significant differences in weight (*t* = 0.474, *p* = 0.636) or BMI (23.53 ± 3.16 vs. 22.17 ± 4.80 kg/m²; *t* = 1.583, *p* = 0.115) between the OP and Con groups.

**Table 1:** Comparison of demographic and laboratory parameters between the OP and Con groups [*x ± s*, n (%)]

Index	OP group (*n* = 75)	Con group (*n* = 140)	t/χ^2^	*p*
Gender (male/female)	9/66	64/76	23.273	< 0.001
Age (year)	67.85 ± 8.59	47.3 ± 14.53	13.007	< 0.001
Height (m)	1.62 ± 0.05	1.66 ± 0.08	–4.892	< 0.001
Weight (kg)	61.81 ± 9.03	61.14 ± 11.38	0.474	0.636
BMI (kg/m^2^)	23.53 ± 3.16	22.17 ± 4.80	1.583	0.115
ALT (U/L)	16.58 ± 9.28	20.18 ± 9.30	–2.746	0.007
AST (U/L)	18.65 ± 4.93	18.15 ± 4.23	1.184	0.239
HDL (mmol/L)	1.31 ± 0.37	1.36 ± 0.26	–1.205	0.231
LDL (mmol/L)	2.88 ± 0.90	2.71 ± 0.61	1.420	0.158
Lumbar T-score	–2.44 ± 0.90	0.02 ± 0.53	–21.735	< 0.001
Hip T-score	–2.39 ± 0.73	–0.01 ± 0.59	–24.025	< 0.001
Vitamin D (nmol/L)	13.56 ± 4.14	15.01 ± 2.87	–2.701	0.008
P (mmol/L)	1.22 ± 0.72	1.22 ± 0.71	–0.265	0.792
Ca (mmol/L)	2.29 ± 0.11	2.29 ± 0.11	–0.026	0.979

#### Laboratory-related index analysis

##### Liver function indicators

Mean ALT levels were significantly lower in the OP group than in the Con group (*t* = − 2.746, *p* = 0.007), whereas AST levels did not differ significantly between the two groups (*t* = 1.184, *p* = 0.239).

##### Lipid metabolism indicators

There were no statistically significant differences in HDL (1.31 ± 0.37 vs. 1.36 ± 0.26 mmol/L; *t* = − 1.205, *p* = 0.231) or LDL levels (2.88 ± 0.90 vs. 2.71 ± 0.61 mmol/L; *t* = 1.420, *p* = 0.158) between the OP and Con groups.

##### Bone health-related indicators

The lumbar T-scores in the OP group (–2.44 ± 0.90) were markedly lower than those in the Con group (0.02 ± 0.53) (*t* = − 21.735, *p* < 0.001). Similarly, hip T-scores also showed a highly significant reduction in the OP group (*t* = − 24.025, *p* < 0.001). Plasma vitamin D levels showed a decreasing trend in the OP group and were significantly lower than those in the Con group (*t* = − 2.701, *p* = 0.008).

##### Mineral metabolism indicators

There were no significant differences in plasma phosphorus (P) and calcium (Ca) levels among the groups.

### Plasma metabolic profiling and multivariate statistical analysis

**Fig. 1:**
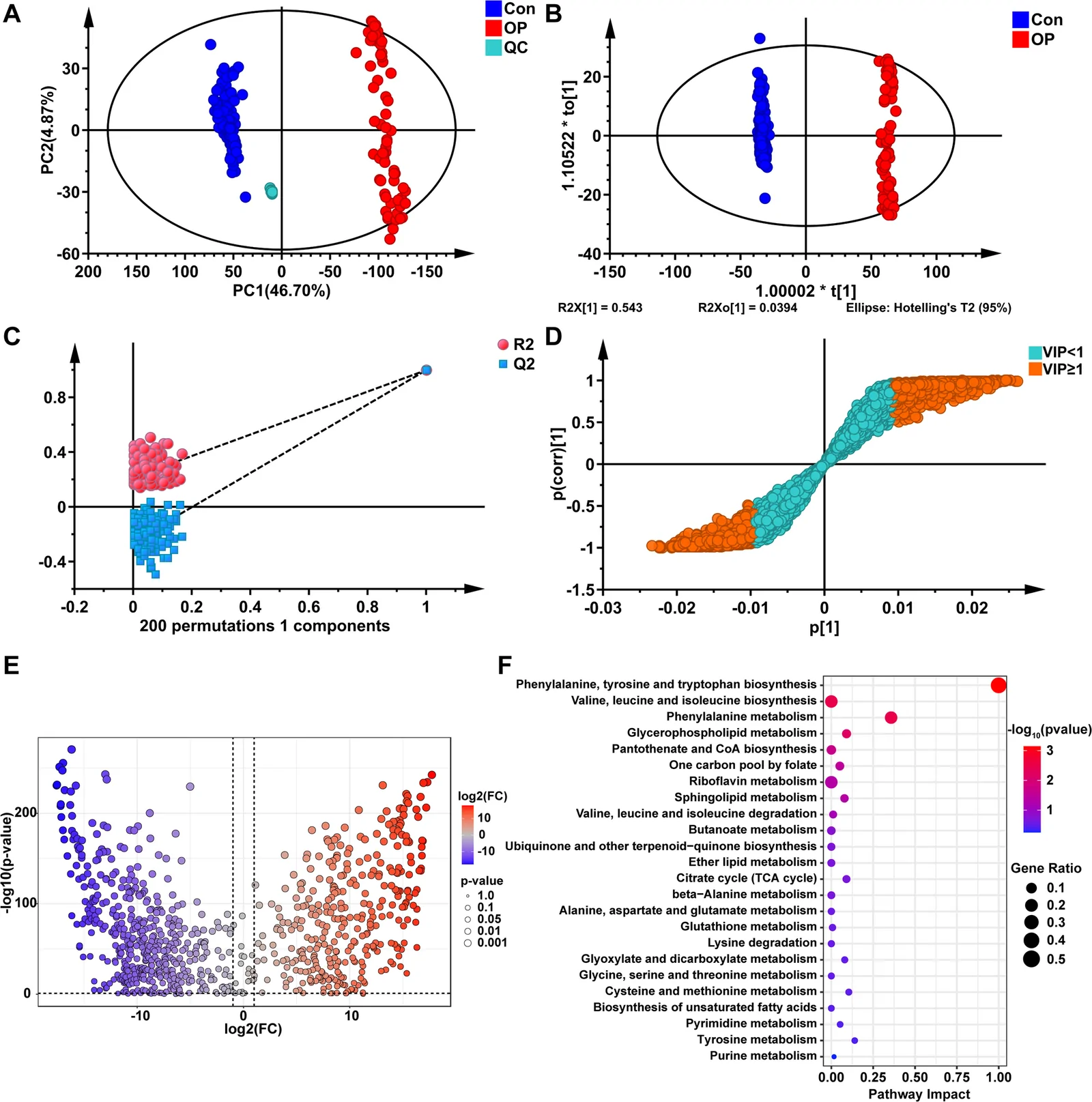
Plasma metabolomics profiling and multivariate statistical analysis in the discovery cohort. **A** PCA score plot between OP and Con groups; **B** OPLS-DA score plot; **C** 200-permutation validation of the OPLS-DA model; **D** S-plot; **E** Volcano plot; **F** Pathway enrichment

Untargeted UHPLC-MS-based metabolic profiling was performed on plasma samples from the discovery cohort, including 75 patients with OP and 140 healthy controls. No additional QC-based signal drift correction methods were applied to the dataset. This decision was justified by the high reproducibility of the pooled QC samples: >95% of detected lipid features showed a coefficient of variation (CV) < 20% across all QC injections, confirming that signal drift over the analytical run was minimal. To account for minor variations in sample loading and injection, all raw lipid intensities were first normalized by the total ion intensity of each sample. PCA revealed a trend toward metabolic separation between the OP and control groups (Fig. 1A). The tight clustering of QC samples further supported the analytical stability and reproducibility of the platform. OPLS-DA was subsequently applied and showed high model performance (R²Y = 0.998, Q² = 0.998) (Fig. 1B). Model robustness was further evaluated by permutation testing (*n* = 200; R² intercept = 0.214, Q² intercept = − 0.254) (Fig. 1C) and CV-ANOVA (*p* < 0.001), suggesting that the model was statistically valid and unlikely to be substantially overfitted. However, given the supervised nature of OPLS-DA, these results should still be interpreted with caution, as supervised models may overestimate group discrimination in clinical metabolomics datasets.

From 11,374 initially detected features, a rigorous filtering and annotation workflow, including retention time alignment, MS/MS spectral matching, and multi-database cross-referencing, identified 61 differential metabolites with high confidence (MSI Level 1 − 2). To further assess whether these metabolic differences were influenced by demographic confounding, age and sex were incorporated as covariates in subsequent adjusted analyses. The results showed that these metabolites remained significantly associated with osteoporosis after adjustment, supporting that the observed metabolic alterations were not solely attributable to demographic differences. In addition, a hierarchical clustering heatmap of the 61 differential metabolites (Figure S1) illustrated the overall metabolic perturbation associated with osteoporosis.

### Identification of potential biomarkers and pathway enrichment analysis

Differential metabolites were prioritized based on a composite threshold of Fold Change (FC) ≥ 1.2 or ≤ 0.833, Variable Importance in Projection (VIP) > 1, and p-value < 0.05, derived from S-plots and volcano plots (Fig. 1D and E). This analysis yielded 61 significantly altered metabolites (Table S1). To elucidate the biological significance of these alterations, Metabolic Pathway Analysis was performed (Fig. 1F). The perturbed metabolites were predominantly enriched in: Amino Acid Metabolism: Including alanine, aspartate, and glutamate metabolism, and branched-chain amino acid (BCAA) degradation. Energy Metabolism: Specifically the Citric Acid Cycle (TCA cycle) and glyoxylate/dicarboxylate metabolism. Lipid Metabolism: Notably glycerophospholipid, sphingolipid, and ether lipid metabolism, alongside the biosynthesis of unsaturated fatty acids. Significantly, 42.6% (26/61) of the identified differential metabolites were lipid species (e.g., phospholipids and sphingolipids). This lipid-centric metabolic signature suggests that dysregulation of lipid homeostasis is a hallmark of the osteoporotic state, providing a robust rationale for subsequent targeted lipidomic validation.

### Targeted lipidomic profiling and identification of differential lipid signatures

Lipid-related features were first observed during the untargeted metabolomics analysis (Figure S1), which was primarily used for global metabolic screening and pathway-level exploration. To further characterize these lipidomic alterations with greater specificity and quantitative resolution, we subsequently performed targeted lipidomics analysis. This targeted approach quantified a total of 1,063 lipid molecular species with MSI Level 2 annotation across both positive and negative ionization modes. Because untargeted metabolomics and targeted lipidomics differ in analytical coverage, annotation confidence, quantification strategy, and feature selection criteria, the final lipid biomarker panel was prioritized mainly based on the targeted lipidomics results, rather than solely on the apparent group differences observed in the untargeted screening analysis.

QC filtering was conducted by excluding lipid features with a missing value rate > 30%, and 897 lipid species were retained after filtering. Ultimately, these 897 qualified lipids were adopted for all downstream bioinformatics and statistical analyses. Significant perturbations were observed in 14 of the 19 lipid subclasses analyzed. Specifically, altered levels of phosphatidylcholine (PC), phosphatidylinositol (PI), free fatty acids (FFA), lysophosphatidic acid (LPA), lysophosphatidylinositol (LPI), lysophosphatidylcholine (LPC), lysophosphatidylethanolamine (LPE), triacylglycerols (TAG), diacylglycerols (DAG), dihydroceramide (DCER), lactosylceramide (LCER), sphingosine-1-phosphate (S1P), sphingomyelin (SM), and ceramide (Cer) were detected in OP patients, indicating widespread remodeling of glycerophospholipid, sphingolipid, and neutral lipid metabolism (Figure S2).

**Fig. 2:**
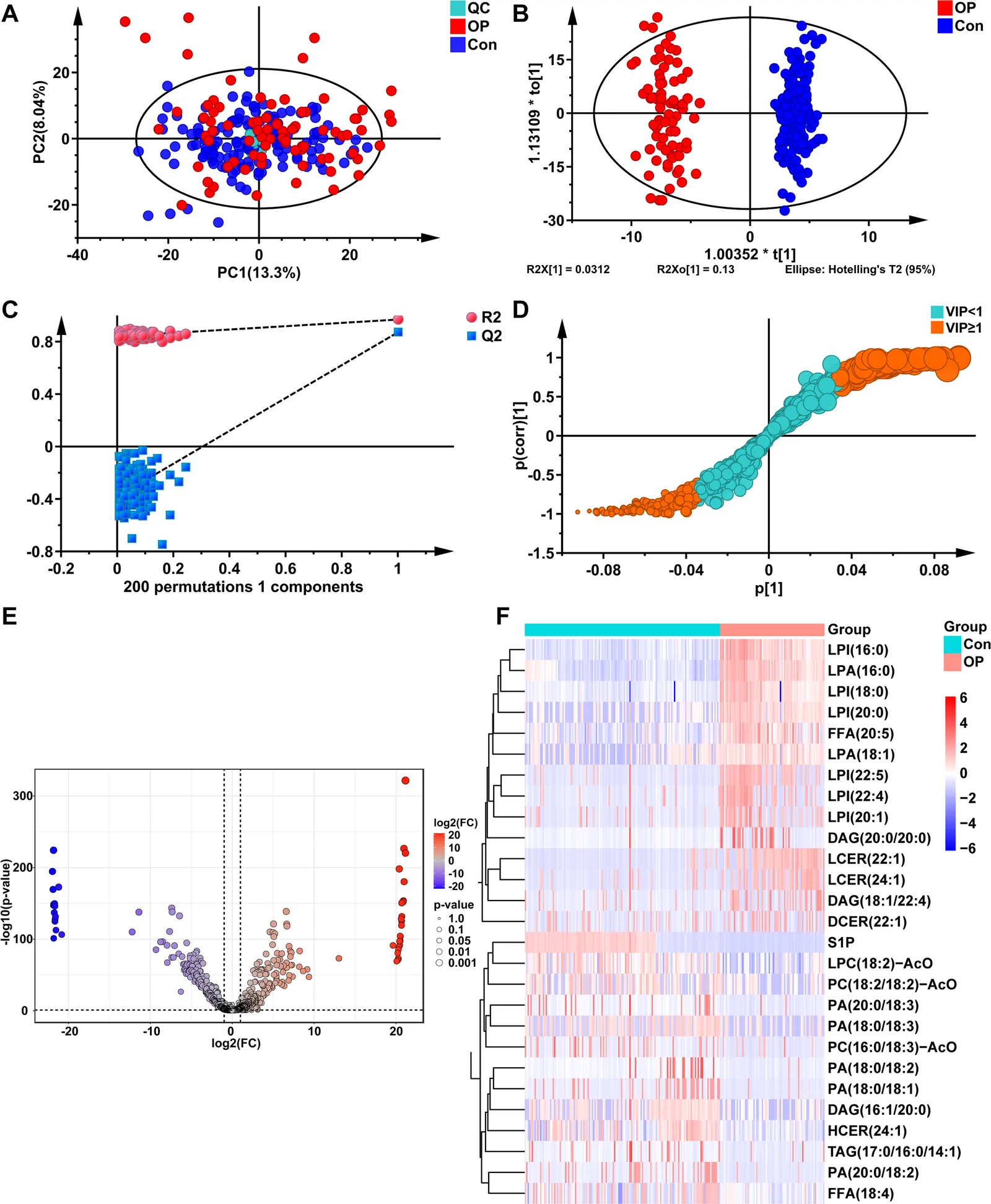
Plasma lipidomics profiling in the discovery cohort. **A** PCA plot between OP and Con groups based on lipidomic data; **B** OPLS-DA score plot; **C** 200-permutation test; **D** S-plot; **E** Volcano plot; **F** Heatmap of 27 differential lipid metabolites

Unsupervised PCA demonstrated clear clustering trends between the OP and control groups (PC1 = 13.3%, PC2 = 8.04%) (Fig. 2A). Supervised OPLS-DA further enhanced group discrimination (R²Y = 0.966, Q² = 0.871) (Fig. 2B), with model robustness supported by permutation testing (*n* = 200; R² = 0.842, Q² = −0.382) (Fig. 2C) and CV-ANOVA (*p* < 0.001).

Using stringent selection criteria (VIP > 1, FC > 1.2 or < 0.833, and FDR-adjusted *p* < 0.05), 27 differential lipids were identified, including 14 upregulated and 13 downregulated species in the OP group (Fig. 2D and E). Importantly, these associations remained statistically significant after adjustment for age and sex, confirming their independence from demographic confounding.

Hierarchical clustering analysis (Fig. 2F) further underscored the distinct lipidomic heterogeneity between the cohorts. The most significant variances were concentrated within the LPA, LPI, PA, Cer, and acylglycerol (DAG/TAG) categories, suggesting that the convergence of multiple lipid-mediated signaling pathways plays a pivotal role in the pathogenesis of osteoporosis.

### Multivariate statistical analysis with demographic covariate correction

To determine whether the observed metabolic separation was influenced by demographic confounding, we conducted covariate-adjusted analyses following established statistical frameworks in metabolomics studies (Chen et al., Diabetes, 2022). Briefly, age and sex were modeled as covariates in a regression-based adjustment for each metabolite/lipid feature, and the resulting covariate-adjusted values were subsequently used for PCA and OPLS-DA. In addition, demographic adjustment was also considered in downstream feature evaluation and model validation, so that the potential influence of age and sex was assessed throughout the analytical workflow rather than only at the visualization stage.

In the discovery cohort, both the adjusted PCA (Figure S3A) and adjusted OPLS-DA (Figure S3C) still showed a trend toward separation between the OP and control groups. However, compared with the unadjusted model, the adjusted OPLS-DA model showed substantially lower performance (R²Y = 0.566, Q² = 0.333), indicating a more conservative estimate of group discrimination after accounting for age and sex. Permutation testing (*n* = 200) yielded a negative Q² intercept of − 0.245 (Figure S3E), suggesting that the adjusted model was unlikely to be substantially overfitted. Detailed statistical parameters for the differential metabolites identified in the discovery cohort, including adjusted fold change (FC), variable importance in projection (VIP) scores, and adjusted *p* values, are summarized in Table S2, thereby providing covariate-adjusted evidence for metabolite prioritization.

In the targeted lipidomics validation phase, similar patterns were observed. The adjusted PCA and OPLS-DA analyses also showed a tendency toward group differentiation (Figure S3B and S3D), and the corresponding permutation test produced a negative Q² intercept of − 0.277 (Figure S3F), further supporting model stability after demographic adjustment. Moreover, in the final diagnostic evaluation, the lipid panel was further assessed using an age- and sex-adjusted regression framework, allowing the robustness of the selected biomarkers to be examined under demographic correction during validation.

Overall, these results suggest that the observed metabolic and lipidomic differences may not be entirely explained by age or sex. However, given the limitations of supervised multivariate modeling and the cross-sectional design, these findings should still be interpreted cautiously and warrant further validation in independent cohorts.

### Validation of the lipidomics-based prediction model

To evaluate the diagnostic performance of lipid biomarkers identified in the discovery cohort, an independent validation cohort (*n* = 62; 20 OP patients and 42 healthy controls) was analyzed. Detailed demographic and clinical characteristics of the study participants are summarized in Table S3. No significant differences in age or sex were observed between groups, ensuring cohort comparability.

**Fig. 3:**
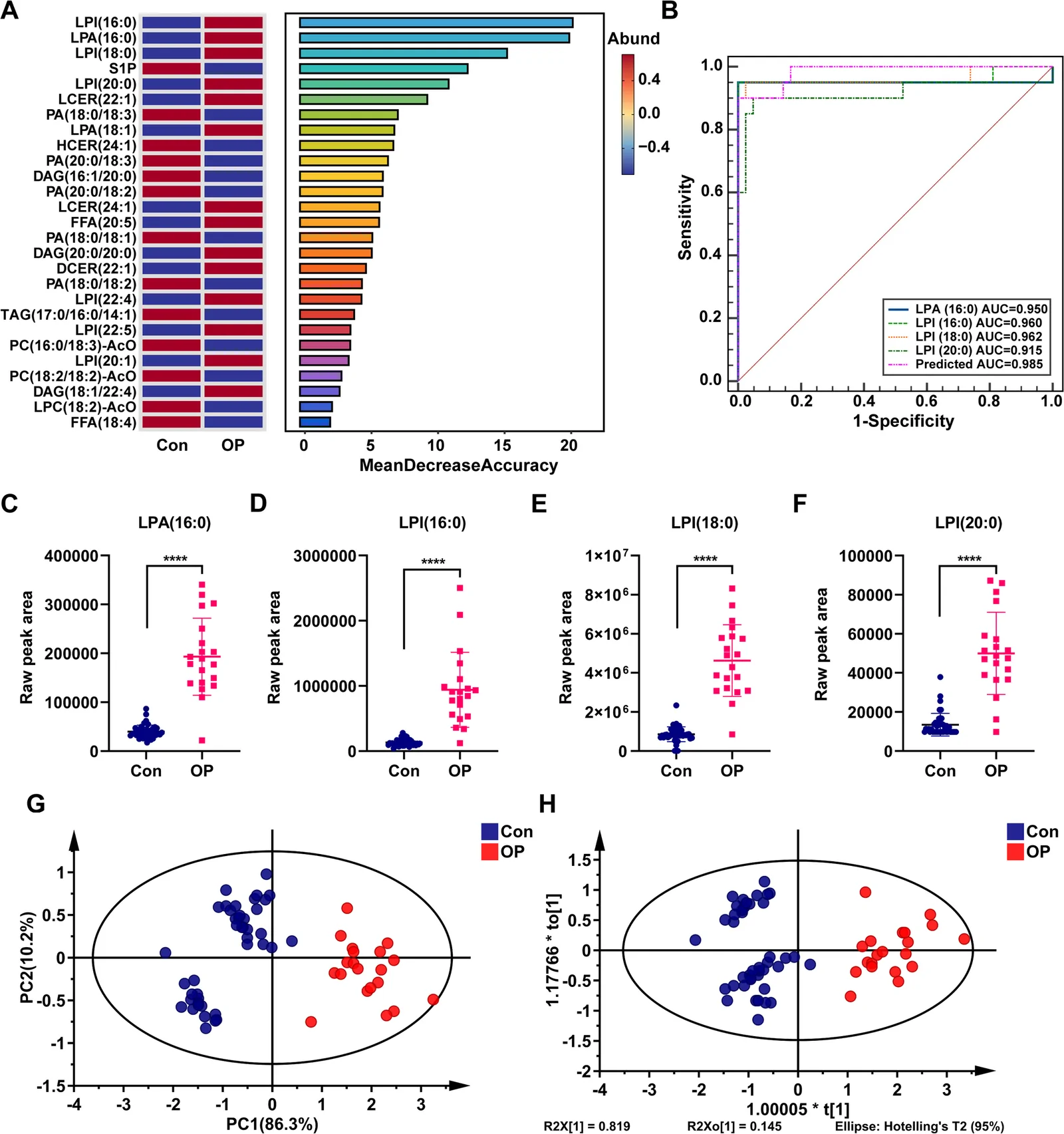
Feature selection and performance of the discovery-phase diagnostic model. **A** Random forest importance ranking; **B** ROC curves of individual lipid biomarkers and the combined unadjusted diagnostic panel (discovery-phase, AUC = 0.985); **C**-**F** The original peak area quantitative comparison of plasma LPA(16:0), LPI(16:0), LPI(18:0), and LPI(20:0) levels between OP and Con groups (*p* < 0.001); **G** PCA plot in the validation cohort; (H) OPLS-DA model

**Fig. 4:**
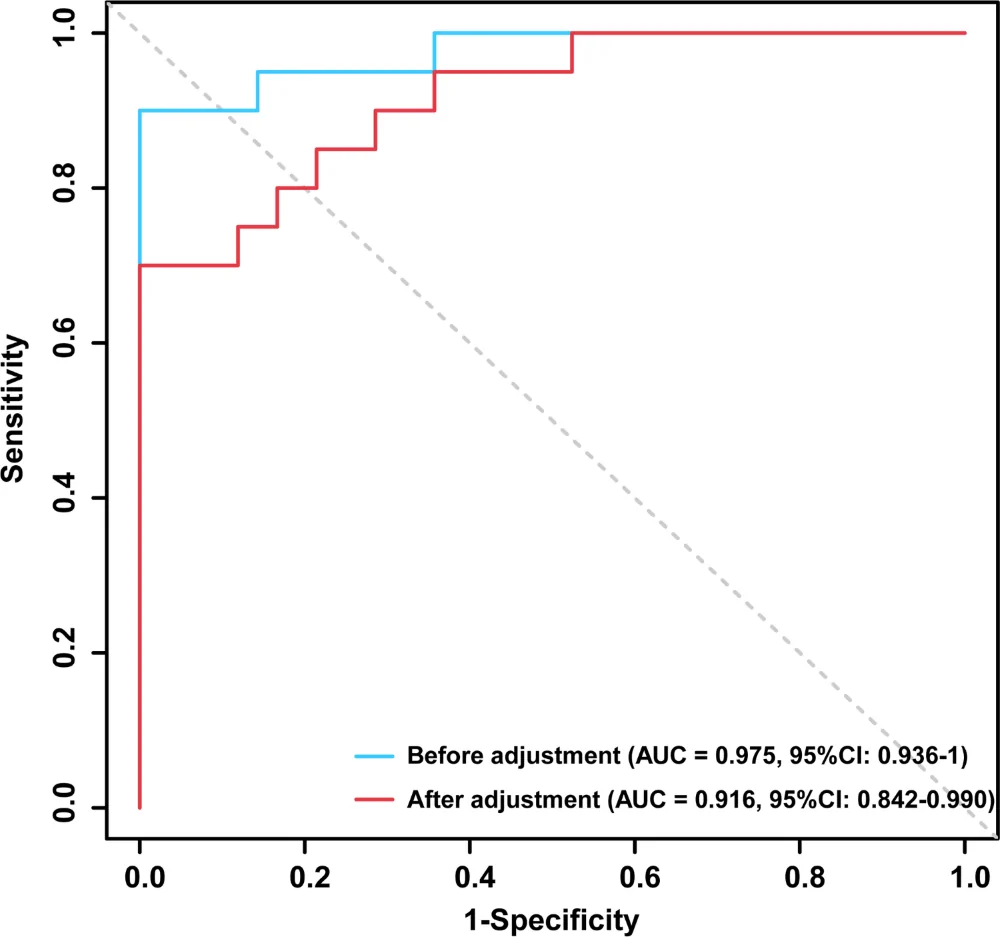
Comparison of model performance before and after adjustment for age and sex

A random forest (RF) algorithm was applied to the top 10% of lipid species ranked by differential abundance to identify the most informative diagnostic features (Fig. 3A). Four lysophospholipids—LPA (16:0), LPI (16:0), LPI (18:0), and LPI (20:0)—were identified as the most discriminative biomarkers. Detailed multivariate statistical parameters for these four lipids, both before and after adjusting for age and sex, are summarized in Table S4. Notably, all four species maintained high VIP scores (> 1.2) and remained highly significant (*p* < 0.001) in the adjusted models. Individual ROC analyses demonstrated high diagnostic performance, with AUC values of 0.950 (95% CI: 0.863–0.989), 0.960 (95% CI: 0.876–0.993), 0.962 (95% CI: 0.880–0.994), and 0.915 (95% CI: 0.817–0.971), respectively. When combined into a multi-biomarker panel, predictive performance was further improved. The optimal lipid panel was identified using LASSO-logistic regression, where the penalty parameter was determined via 10-fold cross-validation to minimize the binomial deviance. To ensure the independence of the diagnostic signature, age and sex were incorporated as mandatory covariates during the model construction process. The detailed configuration of model is summarized in Table S5. Importantly, the integrated panel retained strong discriminative ability after this adjustment, achieving an AUC of 0.916 (95% CI: 0.842–0.990) (Fig. 4). The diagnostic performance of the different models is summarized in Table S6.

Consistent with the discovery cohort, plasma levels of all four lysophospholipids were significantly elevated in OP patients compared to controls (*p* < 0.001) (Figs. 3C-F). Multivariate analyses using PCA and OPLS-DA further demonstrated clear group separation (Figs. 3G-H), with no overlap in the 95% confidence intervals (Hotelling’s T² ellipses), supporting the robustness and reproducibility of the identified biomarker signature.

**Fig. 5:**
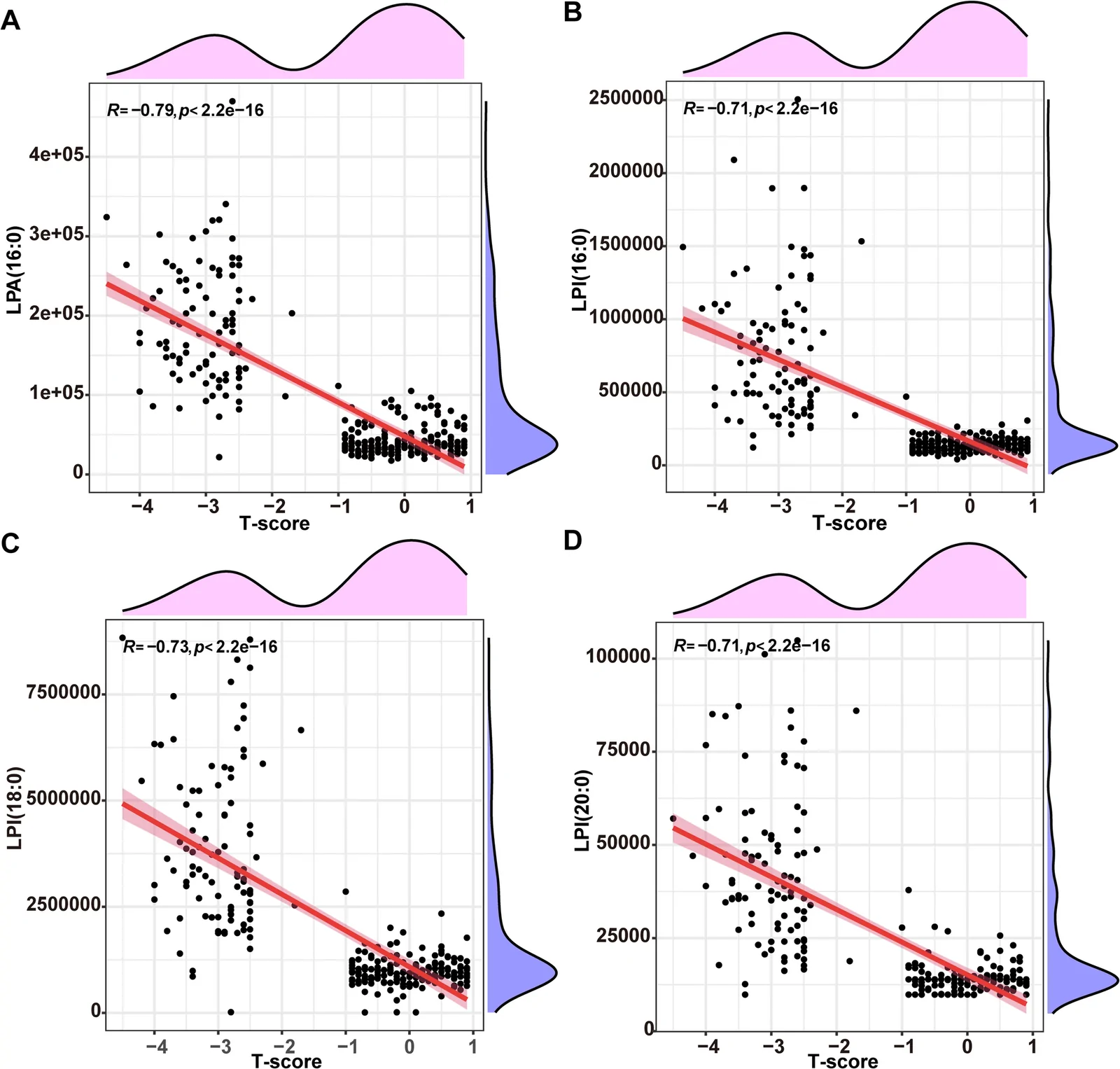
Correlation between key lipid biomarkers and bone mineral density (BMD). **A**-**D** Pearson correlation between LPA(16:0)/LPI(16:0)/LPI(18:0)/LPI(20:0) levels and BMD T-scores

To investigate the relationship between the four lipid biomarkers and bone health status, we performed Pearson correlation analyses between lipid abundance and BMD T-scores. All four metabolites exhibited strong and highly significant negative correlations with T-scores (*p* < 0.001), with correlation coefficients (R) of − 0.79 for LPA(16:0), − 0.71 for LPI(16:0), − 0.73 for LPI(18:0), and − 0.71 for LPI(20:0) (Fig. 5). These findings indicated that higher levels of specific lipid species correlate closely with reduced bone mineral density, supporting their biological plausibility and clinical relevance as diagnostic biomarkers for osteoporosis.

## Discussion

Metabolomics provides a comprehensive overview of small-molecule metabolites and enables the identification of disease-associated biochemical signatures by comparing metabolic profiles between patients and healthy individuals. Metabolites integrate downstream influences of genetic regulation, environmental exposures, and pharmacologic interventions, serving as sensitive indicators of physiological and pathological states. Consequently, metabolomics has increasingly contributed to biomarker discovery, disease stratification, and hypothesis generation in complex metabolic disorders.

Osteoporosis is a complex metabolic bone disorder characterized by reduced BMD and increased fracture risk. Emerging evidence indicates that systemic metabolic alterations, particularly in lipid metabolism, may be associated with the pathophysiology of bone loss. In the present study, we applied an integrated analytical strategy combining untargeted metabolomics, targeted lipidomics, and machine learning approaches to identify plasma metabolic signatures associated with osteoporosis. The results revealed substantial metabolic differences between OP patients and healthy individuals and identified a lipid biomarker panel with strong discriminatory capacity. Importantly, these associations remained significant after adjustment for age and sex, suggesting that the identified lipid species may serve as circulating metabolic indicators associated with osteoporosis.

In this prospective cohort study, we enrolled 95 patients clinically diagnosed with osteoporosis (OP) and 182 healthy controls (Con), performing combined untargeted metabolomics and targeted lipidomics analyses on fasting plasma samples. Untargeted profiling identified 61 significantly altered metabolites, with lipid-related pathways constituting the major component of the metabolic dysregulation. Targeted lipidomics further refined these findings, identifying 27 significantly altered lipid species. Through cross-validation using random forest models and logistic regression, four lysophospholipids, including LPA(16:0), LPI(16:0), LPI(18:0), and LPI(20:0), were validated as robust markers. Their combined diagnostic performance (adjusted AUC = 0.916) demonstrated robust discriminatory ability and consistent reproducibility in an external validation cohort, highlighting their potential as candidate biomarkers for OP risk assessment. However, it should be emphasized that these findings are primarily associative and hypothesis-generating; further prospective and mechanistic studies are warranted to establish definitive causal links between these lipid species and bone metabolism.

### Baseline phenotypic characteristics associated with osteoporosis

Demographic analysis of the discovery cohort revealed that patients in the osteoporosis (OP) group were significantly older, shorter in stature, and exhibited higher BMIs compared to healthy controls, consistent with documented epidemiological trends. Notably, a significant imbalance in age and sex distribution was observed between the two groups. While age remains the most profound risk factor for bone mass deterioration, and postmenopausal estrogen deficiency significantly accelerates bone loss, such demographic disparities could potentially confound the identification of disease-specific metabolic signatures. To mitigate these confounding effects and ensure the identified biomarkers were independently associated with OP pathophysiology, age and sex were incorporated as fixed covariates in all subsequent multivariable statistical analyses and diagnostic modeling.

Regarding laboratory indicators, the OP group presented a lipid profile characteristic of dyslipidemia, specifically marked by decreased HDL and elevated LDL levels. These findings further support the hypothesis that systemic lipid metabolism disorders contribute to the imbalance of bone homeostasis. As anticipated, the OP group exhibited significantly reduced lumbar spine T-scores, while hip T-scores and mineral metabolism indicators (e.g., calcium and phosphorus) showed no significant differences. This suggests that early-stage bone loss, particularly within trabecular-rich regions, may characterize this cohort. In summary, age-related physiological decline, dyslipidemia, and trabecular bone deterioration collectively form the metabolic basis of osteoporosis in this clinical setting (Qadir et al., 2020; Wei et al., 2022; Ren et al., 2024; Tamimi et al., 2020).

Nevertheless, several limitations should be acknowledged when interpreting these findings. The discovery cohort was not formally matched for age and sex, and detailed information on metabolic comorbidities and long-term medication use was unavailable, leaving the possibility of residual confounding. In addition, the limited number of relatively younger OP patients prevented evaluation of whether the identified lipid alterations occur before age-related metabolic remodeling becomes prominent. Therefore, these lipid changes should be interpreted as OP-associated metabolic alterations rather than definitive osteoporosis-specific biomarkers. Further studies with larger, well-matched cohorts and comprehensive clinical data are needed to validate these findings.

### Plasma metabolic alterations associated with osteoporosis

Untargeted UHPLC-MS effectively distinguished the metabolomes of OP patients from healthy individuals, as demonstrated by PCA and OPLS-DA. Pathway enrichment analysis of 61 differentially expressed metabolites revealed profound systemic metabolic alterations in OP, including amino acid metabolism, energy metabolism, and-most notably-lipid metabolism.

Firstly, widespread perturbations in amino acid pathways—including alanine, aspartate, and glutamate metabolism, serine/glycine/threonine metabolism, and branched-chain amino acid (BCAA) degradation—suggest abnormal protein turnover and skewed energy substrate utilization. The disruption of BCAA catabolism is particularly noteworthy given its established role in muscle-bone crosstalk. Within the context of the ‘bone-muscle unit,’ altered BCAA metabolism may be linked to sarcopenia (Lee et al., 2025) and accelerated bone loss potentially exacerbated by reduced mechanical loading in OP (Liang et al., 2025; Lin et al., 2018).

Secondly, significant alterations in energy metabolism were observed, specifically involving the TCA cycle and glyoxylate/dicarboxylate metabolism. These findings imply a potential impairment in mitochondrial oxidative capacity and dysregulation of anaplerotic pathways (He et al., 2025). Such metabolic constraints might compromise cellular energy availability, thereby potentially disrupting the osteoblast-osteoclast coupling essential for balanced bone remodeling (Chen et al., 2025; Da et al., 2021; Lucas et al., 2018).

Most prominently, lipid metabolism emerged as a central metabolic hallmark of OP. Of the 61 differential metabolites, 26 were lipid-related species, including lysophospholipids, sphingolipids, and acylcarnitines. Alterations in glycerophospholipid, sphingolipid, and ether lipid pathways highlighted extensive systemic lipid remodeling (Xiao et al., 2024). Given the fundamental roles of phospholipids in membrane integrity and sphingolipids in signal transduction, these disturbances are hypothesized to influence osteoblast/osteoclast differentiation and activity (Chu et al., 2024; Leng et al., 2024; Zhang et al., 2025).

Furthermore, elevated acylcarnitines likely reflect dysregulated β-oxidation and mitochondrial dysfunction (Gnoni et al., 2020). Overall, these findings suggest that osteoporosis is a systemic metabolic disorder characterized by coordinated alterations across multiple metabolic networks, with lipid dysregulation serving as a core signature of the disease state.

### Dysregulation of plasma lipid metabolism in osteoporosis

Targeted lipidomics revealed widespread alterations in multiple lipid species in osteoporosis (OP), involving phospholipid, sphingolipid, and glycerolipid metabolism. These findings suggest substantial remodeling of lipid metabolic pathways in OP. However, given the observational and cross-sectional design of the present study, the identified lipid alterations should be interpreted as OP-associated metabolic changes rather than evidence of direct causal involvement in disease pathogenesis. It remains unclear whether these lipid changes contribute to bone loss, arise as a consequence of osteoporosis, or reflect broader metabolic alterations associated with aging, sex, metabolic status, or other comorbidities.

Among the altered lipid species, lysophosphatidic acids (LPAs), particularly LPA(16:0) and LPA(18:1), were significantly increased in OP patients. Previous studies have shown that LPA can activate LPA1-mediated Rho/ROCK and NF-κB signaling pathways (Tsukahara & Ueda, 2016), which have been implicated in osteoclast-related signaling and bone remodeling processes (Drzazga et al., 2017; Lin et al., 2024; Zeng et al., 2016). Similarly, lysophosphatidylinositols (LPIs), including LPI(16:0), LPI(18:0), and LPI(20:0), have been reported as endogenous agonists of GPR55 (Alhouayek et al., 2018). GPR55 signaling has been associated with Ca²⁺/ERK pathway activation and inflammatory responses (Odongo et al., 2025), suggesting a potential link between altered LPI abundance and pathways relevant to bone metabolism. Nevertheless, the present study did not directly evaluate these signaling mechanisms, and therefore no causal conclusions can be drawn.

Increased phosphatidic acid (PA) levels may reflect alterations in phospholipid turnover, phospholipase D activity, or downstream mTORC1-related signaling, which have previously been linked to autophagy and osteoclast-related processes (Borel et al., 2020; Zhang et al., 2025). Elevated ceramides (Cers), particularly long-chain species such as LCER(22:1) and HCER(24:1), may indicate broader disturbances in sphingolipid metabolism. Previous studies have associated ceramides with insulin resistance, mitochondrial dysfunction, oxidative stress, impaired osteogenic differentiation, and alterations in Akt-, Runx2-, and RANKL/OPG-related pathways (Khayrullin et al., 2019; Yahiro et al., 2020; Yang et al., 2024). However, whether these ceramide changes are directly involved in osteoporosis development or simply reflect secondary metabolic remodeling remains uncertain.

Altered diacylglycerol (DAG) and triacylglycerol (TAG) species may further reflect disturbances in lipid storage, mobilization, and utilization. These changes may be associated with altered inflammatory signaling, bone marrow adiposity, or broader metabolic dysfunction (Stelzner et al., 2016). However, because these biological processes were not directly assessed in the present study, such interpretations should be regarded as speculative.

Overall, the lipid alterations identified in OP were associated with pathways potentially relevant to bone remodeling, inflammation, and lipid metabolism. However, the present findings are primarily associative and hypothesis-generating. These lipid signatures may serve as candidate metabolic biomarkers of OP, but further longitudinal studies and mechanistic investigations are required to determine whether they represent causal mediators, downstream consequences of bone loss, or broader metabolic correlates of osteoporosis.

### Diagnostic characteristics of differential lipids in the multi-metabolite model

The Random Forest model was used during the discovery phase for feature ranking and preliminary discrimination analysis, and its AUC of 0.985 represents the performance of an unadjusted discovery-phase model. To establish a more clinically relevant and robust diagnostic framework, we subsequently constructed LASSO-logistic regression models incorporating demographic covariates.

Among the identified lipid biomarkers, four lipid species—LPA(16:0), LPI(16:0), LPI(18:0), and LPI(20:0)—showed strong individual discriminatory performance, with AUC values ranging from 0.915 to 0.962 across the discovery and validation cohorts. When combined, these four lipids achieved an unadjusted AUC of 0.975, indicating substantial diagnostic potential. However, considering the baseline demographic differences between groups, particularly age and sex, we further established a covariate-adjusted LASSO-logistic regression model. After adjustment, the four-lipid panel retained robust diagnostic performance, yielding a final AUC of 0.916 (95% CI: 0.842–0.990). Because this model accounted for demographic confounding, it was considered the primary proposed diagnostic framework in the present study. In addition, all four lipids were negatively correlated with bone mineral density T-scores, further supporting their potential clinical relevance.

Previous human metabolomics and lipidomics studies have reported altered lipid metabolism in OP or low-BMD cohorts, particularly involving glycerophospholipids, sphingolipids, triglycerides, phosphatidylinositol, phosphatidic acid, phosphatidylcholine, lysophosphatidylcholine, lysophosphatidic acid, and related lipid pathways. These findings support the involvement of lipid metabolic remodeling in OP. Notably, increased LPA(16:0) has been reported in a previous human postmenopausal osteoporosis lipidomics study, which is directionally consistent with the increase observed in the present study (Lee et al., 2020). However, the specific LPI species identified here, including LPI(16:0), LPI(18:0), and LPI(20:0), have not been consistently reported as OP-associated biomarkers in previous human OP or low-BMD cohorts. Therefore, our study extends previous work by identifying a lysophospholipid-related lipid signature associated with OP. Nevertheless, differences in study population, sample type, analytical platform, lipid coverage, and statistical strategy may limit direct comparison across studies, and further validation in larger cohorts is needed.

From a mechanistic perspective, these lipid alterations may be associated with pathways involved in bone remodeling and inflammatory regulation. Previous studies have suggested that LPA(16:0) may activate LPAR1-mediated Rho/ROCK and NF-κB signaling, potentially promoting NFATc1-related osteoclast differentiation and bone resorption (Zhu et al., 2018). Similarly, LPI species have been proposed as endogenous GPR55 agonists and may be associated with Ca²⁺/ERK signaling pathways (Odongo et al., 2025), which could influence osteoblast differentiation and inflammatory responses (Kurano et al., 2021). In addition, differences in acyl chain composition may affect receptor affinity and signaling stability, with LPI(16:0) and LPI(18:0) showing relatively stronger associations with chronic metabolic dysregulation.The signaling effects of LPA and LPI likely converge downstream on the MAPK/Akt pathway (Riaz et al., 2016; Zhang et al., 2020), synergistically elevating the RANKL/OPG ratio and shifting bone metabolism toward a pro-ressorptive state. Furthermore, enhanced sPLA_2_ activity observed in OP may accelerate the production of both LPA and LPI, potentially forming a feedforward loop that exacerbates bone deterioration (Law et al., 2019).

In conclusion, these four lysophospholipids serve as reliable biomarkers for early OP detection. Their direct involvement in osteoclast activation and osteoblast suppression underscores their potential as biomarker-guided targets for precision intervention and therapeutic monitoring (Table 2).

**Table 2:** Comparison of previous human osteoporosis-related metabolomics/lipidomics studies and the present study

Study	Population	Sample type	Platform	Main findings	Overlap with current study
(Cabrera et al., 2018)	Low-BMD menopausal women	Plasma	LC-MS metabolomics/lipidomics	Altered glycerophospholipids, PI, PA, sphingolipids	Supports lipid remodeling, no direct LPI overlap
(Lee et al., 2020)	Postmenopausal osteoporosis	Plasma	Lipoprotein lipidomics	Increased LPA(16:0), altered PC, TG, ceramide species	LPA(16:0) directionally consistent
(Aleidi et al., 2021)	Osteopenia/osteoporosis subjects	Serum	Untargeted metabolomics	Altered amino acid and lipid metabolism pathways	Supports metabolic dysregulation in OP; no direct overlap with identified lipid species
(Aleidi et al., 2022)	Low BMD/osteoporosis	Serum	Untargeted lipidomics	Altered phospholipid and sphingolipid metabolism	Supports lipidomic alterations; no specific LPA/LPI panel reported
(Kou et al., 2022)	Postmenopausal osteoporosis patients	Serum	LC-MS/GC-MS metabolomics	Disturbed lipid, amino acid, and energy metabolism	Consistent pathway-level findings
Present study	Osteoporosis patients	Plasma	Untargeted metabolomics + targeted lipidomics	Increased LPA(16:0), LPI(16:0), LPI(18:0), and LPI(20:0)	Identified a lysophospholipid-related lipid signature associated with OP

## Conclusion

By integrating multi-stage metabolomics with machine learning, this study identified a four-lysophospholipid panel—LPA(16:0), LPI(16:0), LPI(18:0), and LPI(20:0)—as a robust metabolic signature of osteoporosis (OP). The diagnostic model maintained a high AUC of 0.916 (95% CI: 0.842–0.990) after stringent adjustment for age and sex, underscoring its independent predictive value. These findings support a role for systemic lipid dysregulation in OP and suggest a potential non-invasive approach for early risk assessment. However, the study’s cross-sectional, single-center design precludes causal inference, necessitating further multi-center longitudinal validation and functional mechanistic studies.

## Supplementary information

Below is the link to the electronic supplementary material.

Supplementary Material 1

Supplementary Material 2

Supplementary Material 3

Supplementary Material 4

Supplementary Material 5

Supplementary Material 6

Supplementary Material 7

Supplementary Material 8

Supplementary Material 9

Supplementary Material 10

## Data Availability

The processed metabolomics and lipidomics datasets, feature tables, and associated metadata supporting the findings of this study are publicly available in the Figshare repository at DOI: https://doi.org/10.6084/m9.figshare.31810990.

## Abbreviations

ALT: Alanine aminotransferase
AST: Aspartate aminotransferase
AUC: Area under the curve
BMD: Bone mineral density
BMI: Body mass index
DXA: Dual-energy X-ray absorptiometry
ESI: Electrospray ionization
FC: Fold change
FDR: False discovery rate
HCD: Higher-energy collisional dissociation
HDL-C: High-density lipoprotein cholesterol
HESI: Heated electrospray ionization
KEGG: Kyoto Encyclopedia of Genes and Genomes
LDL-C: Low-density lipoprotein cholesterol
LPA: Lysophosphatidic acid
LPI: Lysophosphatidylinositol
MRM: Multiple reaction monitoring
OP: Osteoporosis
OPLS-DA: Orthogonal partial least squares discriminant analysis
PCA: Principal component analysis
QC: Quality control
RF: Random forest
ROC: Receiver operating characteristic
UHPLC-MS: Ultra-performance liquid chromatography-mass spectrometry
VIP: Variable importance plots
β-CTX: Type I collagen
