# Supplementary material for: Integration of metabolomics and machine learning algorithm for discovery of early diagnostic biomarkers of osteoporosis
Source: Metabolomics. 2026 Jul 14;22(4):126. doi: 10.1007/s11306-026-02506-5 (PMC13369700; doi:10.1007/s11306-026-02506-5)
Supplement: Supplementary file 1 — Supplementary Material 1 [file 11306_2026_2506_MOESM1_ESM.docx]

**Figure S1. Heatmap of differential metabolites**
