# Supplementary figures and images for: Integration of metabolomics and machine learning algorithm for discovery of early diagnostic biomarkers of osteoporosis
Source: Metabolomics. 2026 Jul 14;22(4):126. doi: 10.1007/s11306-026-02506-5 (PMC13369700; doi:10.1007/s11306-026-02506-5)

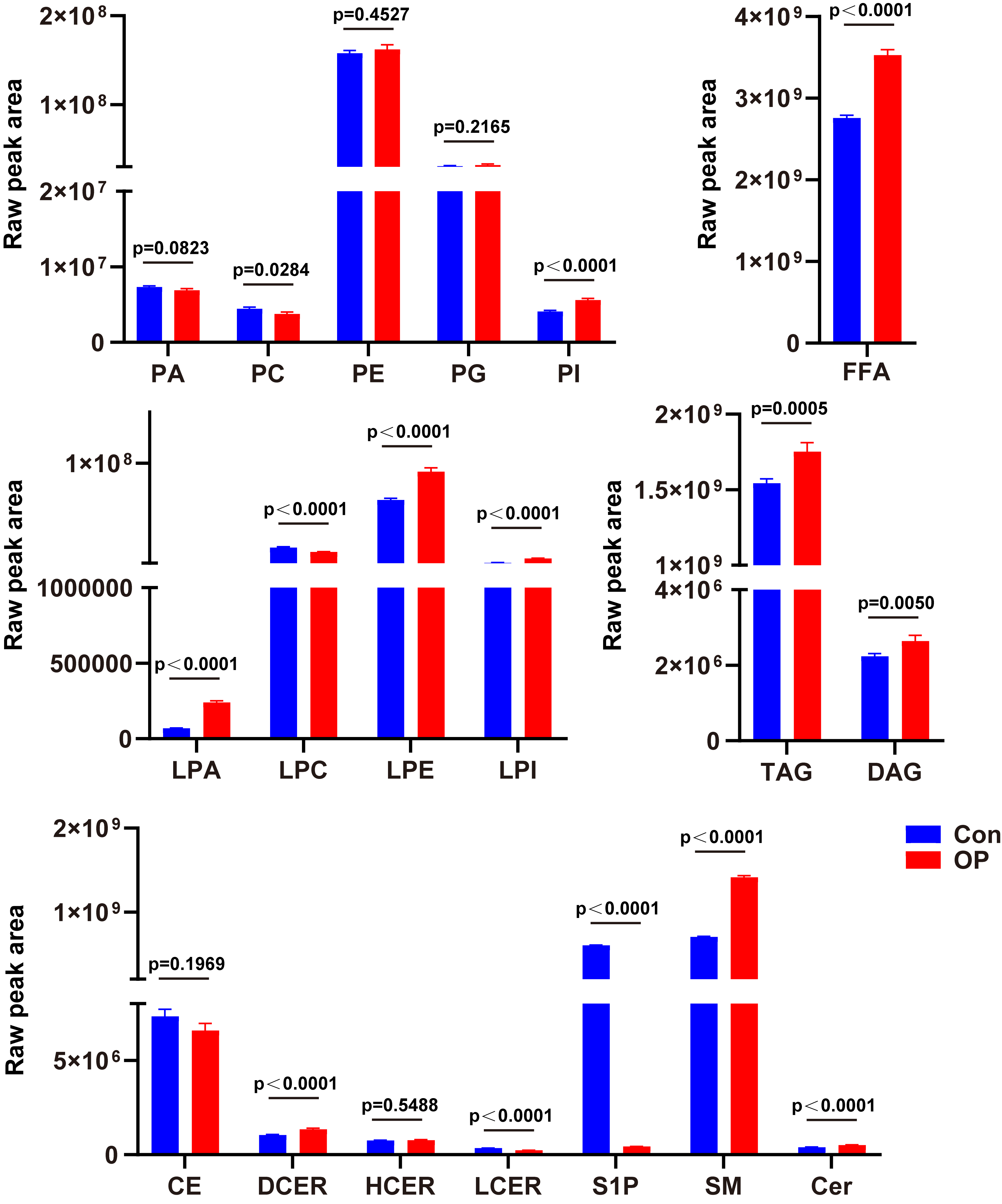


**Figure S2. The content levels of 19 lipid subclasses in serum**

Supplement: Supplementary file 2 — Supplementary Material 2 [file 11306_2026_2506_MOESM2_ESM.docx]
