# Supplementary material for: Integration of metabolomics and machine learning algorithm for discovery of early diagnostic biomarkers of osteoporosis
Source: Metabolomics. 2026 Jul 14;22(4):126. doi: 10.1007/s11306-026-02506-5 (PMC13369700; doi:10.1007/s11306-026-02506-5)
Supplement: Supplementary file 3 — Supplementary Material 3 [file 11306_2026_2506_MOESM3_ESM.docx]

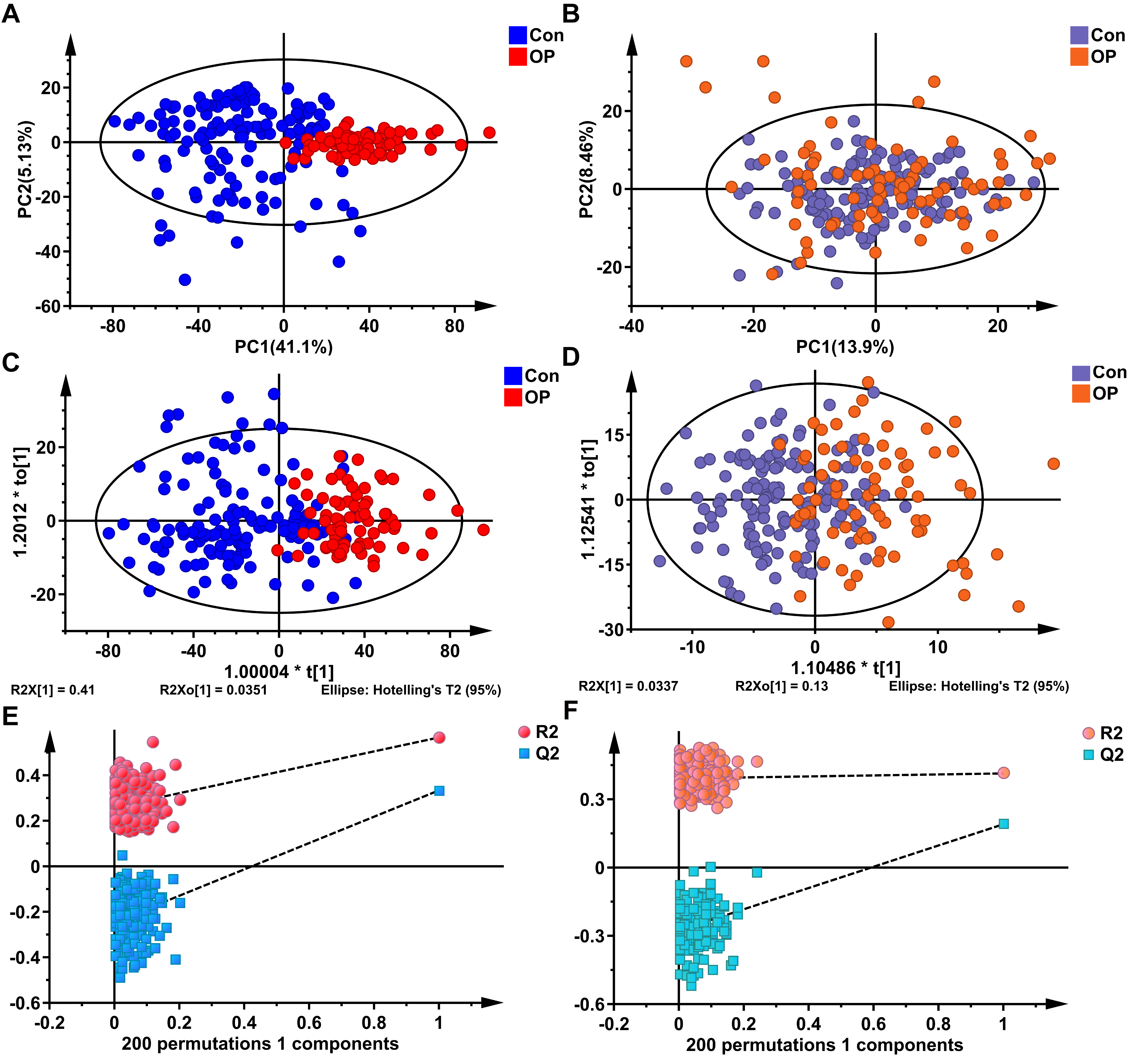


**Figure S3.** **Multivariate Statistical Analysis of Plasma Metabolomics and Lipidomics After Covariate Adjustment**

(A, C, E) Untargeted Metabolomics PCA, OPLS-DA, and Permutation test;

(D, B, F) Targeted Lipidomics PCA, OPLS-DA, and Permutation tes
