# Supplementary material for: Integration of metabolomics and machine learning algorithm for discovery of early diagnostic biomarkers of osteoporosis
Source: Metabolomics. 2026 Jul 14;22(4):126. doi: 10.1007/s11306-026-02506-5 (PMC13369700; doi:10.1007/s11306-026-02506-5)
Supplement: Supplementary file 4 — Supplementary Material 4 [file 11306_2026_2506_MOESM4_ESM.docx]

**Supplymentary Methods**

**Data Preprocessing and Statistical Analysis For Untargeted Metabolomics**

The raw metabolomics data underwent a rigorous preprocessing pipeline to ensure the reliability of downstream statistical analyses. Initially, metabolic features were extracted and converted to a numerical format. Missing values were imputed using the median abundance of the respective metabolite across all samples; to avoid numerical bias, features with a median of zero were replaced with a minimum detectable value (one-half of the minimum non-zero value). To correct for heteroscedasticity and eliminate magnitude effects, the data were subjected to log_2_ transformation followed by Z-score normalization. A secondary filtration step was performed post-standardization to ensure the removal of any residual constant variables. The final preprocessed data matrix was utilized for Principal Component Analysis (PCA) and subsequent biomarker identification.

**Data Preprocessing and Statistical Analysis For Pseudo-targeted Lipidomics**

Lipidomics data processing and normalization. Lipidomics data were initially processed using SCIEX OS for peak integration. Subsequent preprocessing was performed in MetaboAnalyst 6.0, including missing value imputation, sum normalization, log_2_ transformation, and Z-score normalization. Specifically, raw peak intensities were first normalized to the total ion intensity of each sample to correct for differences in sample loading, injection volume, and minor signal variation across analytical runs. The normalized data were then log_2_-transformed to reduce right-skewness and stabilize variance across the wide dynamic range of lipid abundances. Prior to multivariate statistical analyses, each lipid feature was auto-scaled by mean-centering and division by its standard deviation, ensuring that lipids with different absolute abundances and ionization efficiencies contributed comparably to the models. Multivariate analyses, including PCA and OPLS-DA, were conducted using SIMCA 14.1. To ensure robust biomarker selection, both univariate and multivariate criteria were applied, and lipid species were considered significant only when they simultaneously met the following thresholds: VIP ≥ 1, FC > 1.2 or < 0.833, and FDR-adjusted *p* < 0.05.

**Quality control procedures**

For non-targeted metabolomics, pooled QC samples were analyzed every 10 study samples, resulting in a total of 48 QC injections. The majority of retained features showed QC CV values below 30%, indicating acceptable analytical reproducibility. For targeted lipidomics, pooled QC samples were also injected every 10 study samples, with a total of 10 QC injections. Most retained lipid features showed QC CV values below 30%, supporting instrument stability and data reliability during lipidomic analysis. These QC results support the analytical robustness of both the non-targeted metabolomics and targeted lipidomics measurements.

**Machine Learning Model Construction and Validation**

To evaluate the diagnostic potential of the candidate lipid biomarkers, a Random Forest (RF) classification model was constructed using the discovery cohort. RF is a robust ensemble learning algorithm that aggregates multiple decision trees, making it particularly effective for handling high-dimensional metabolomics data and capturing non-linear biological relationships.Prior to model training, lipid intensities underwent log_2_ transformation and Z-score normalization to mitigate data heteroscedasticity. The RF model was implemented using the randomforest package in R. To ensure optimal performance and reproducibility, hyperparameters were tuned via a grid search, specifically: the number of trees (*ntree* = 500) and the number of variables randomly sampled at each split (*mtry* = 3, based on $\surd p$, where p = 10 biomarkers). The final configuration was selected by minimizing the out-of-bag (OOB) classification error rate.To enhance model stability and prevent overfitting, 10-fold cross-validation was performed within the discovery cohort. The dataset was partitioned into ten subsets, with each subset iteratively serving as a validation set while the remaining nine were used for training. Feature contribution was quantified using the Mean Decrease in Accuracy and Mean Decrease in Gini Index, allowing for the identification of the most informative lipid species driving the classification.Finally, the predictive robustness of the optimized RF model was rigorously evaluated in an independent validation cohort. Model discrimination was quantified via Receiver Operating Characteristic (ROC) curve analysis, with the area under the curve (AUC), sensitivity, and specificity calculated along with their respective 95% confidence intervals (CI) to ensure statistical transparency.
