# Supplementary material for: Integration of metabolomics and machine learning algorithm for discovery of early diagnostic biomarkers of osteoporosis
Source: Metabolomics. 2026 Jul 14;22(4):126. doi: 10.1007/s11306-026-02506-5 (PMC13369700; doi:10.1007/s11306-026-02506-5)
Supplement: Supplementary file 5 — Supplementary Material 5 [file 11306_2026_2506_MOESM5_ESM.docx]

**Table S1.** **Information on 61 differential metabolites**

| **NO.** | **RT [min]** | **Name** | **Formula** | **Calc. MW** | ***m/z*** | **Mass error**  **(ppm)** | **Adduction** | **HMDB** | **Confidence**  **level** |
| --- | --- | --- | --- | --- | --- | --- | --- | --- | --- |
| 1 | 0.69 | L-Lysine**^*^** | C_6_H_14_N_2_O_2_ | 146.1056 | 147.1129 | 0.42 | [M+H]^+^ | HMDB0028956 | Level 1 |
| 2 | 0.69 | Pipecolic acid | C_6_H_11_NO_2_ | 129.0791 | 130.0863 | 0.63 | [M+H]^+^ | HMDB0001557 | Level 2 |
| 3 | 0.93 | Valine | C_5_H_11_NO_2_ | 117.0790 | 118.0863 | -0.02 | [M+H]^+^ | HMDB0000883 | Level 2 |
| 4 | 1.00 | Glycerophosphocholine**^*^** | C_8_H_20_NO_6_P | 257.1030 | 258.1102 | 0.51 | [M+H]^+^ | HMDB0000086 | Level 1 |
| 5 | 1.01 | Proline**^*^** | C_5_H_9_NO_2_ | 115.0633 | 116.0706 | 0.07 | [M+H]^+^ | HMDB0251529 | Level 1 |
| 6 | 1.02 | Choline**^*^** | C_5_H_13_NO | 103.0997 | 104.1070 | 0.26 | [M+H]^+^ | HMDB0000097 | Level 1 |
| 7 | 1.05 | DL-Carnitine**^*^** | C_7_H_15_NO_3_ | 161.1053 | 162.1126 | 0.76 | [M+H]^+^ | HMDB0000062 | Level 1 |
| 8 | 1.27 | Hypoxanthine**^*^** | C_5_H_4_N_4_O | 136.0386 | 137.0458 | 0.28 | [M+H]^+^ | HMDB0000157 | Level 1 |
| 9 | 1.27 | Methionine**^*^** | C_5_H_11_NO_2_S | 149.0511 | 150.0584 | 0.14 | [M+H]^+^ | HMDB0000696 | Level 1 |
| 10 | 1.27 | Prolylleucine | C_11_H_20_N_2_O_3_ | 228.1474 | 229.1547 | 0.20 | [M+H]^+^ | HMDB0253028 | Level 2 |
| 11 | 1.31 | L-Pyroglutamic acid | C_5_H_7_NO_3_ | 129.0427 | 130.0499 | 0.66 | [M+H]^+^ | HMDB0000267 | Level 2 |
| 12 | 1.35 | Uracil | C_4_H_4_N_2_O_2_ | 112.0273 | 113.0346 | 0.14 | [M+H]^+^ | HMDB0000300 | Level 2 |
| 13 | 1.43 | Uric acid | C_5_H_4_N_4_O_3_ | 168.0292 | 167.0220 | 5.36 | [M-H]^-^ | HMDB0000300 | Level 2 |
| 14 | 1.45 | Citric acid | C_6_H_8_O_7_ | 192.0274 | 191.0201 | 1.86 | [M-H]^-^ | HMDB0000094 | Level 2 |
| 15 | 1.57 | 3-Hydroxybutyric acid | C_4_H_8_O_3_ | 104.0476 | 103.0403 | 2.46 | [M-H]^-^ | HMDB0000011 | Level 2 |
| 16 | 1.66 | L-Tyrosine**^*^** | C_9_H_11_NO_3_ | 181.0739 | 182.0812 | 0.07 | [M+H]^+^ | HMDB0000158 | Level 1 |
| 17 | 1.67 | Propionylcarnitine | C_10_H_19_NO_4_ | 217.1314 | 218.1387 | 0.06 | [M+H]^+^ | HMDB0000824 | Level 2 |
| 18 | 1.71 | L-Norleucine | C_6_H_13_NO_2_ | 131.0946 | 132.1019 | -0.05 | [M+H]^+^ | HMDB0001645 | Level 2 |
| 19 | 2.73 | L-Phenylalanine**^*^** | C_9_ H_11_NO_2_ | 165.0790 | 166.0863 | 0.10 | [M+H]^+^ | HMDB0000159 | Level 1 |
| 20 | 3.05 | 6-Methylquinoline | C_10_H_9_N | 143.0735 | 144.0808 | 0.19 | [M+H]^+^ | HMDB0033115 | Level 2 |
| 21 | 3.05 | trans-3-Indoleacrylic acid | C_11_H_9_NO_2_ | 187.0632 | 188.0705 | -0.48 | [M+H]^+^ | HMDB0000734 | Level 2 |
| 22 | 3.05 | DL-Tryptophan | C_11_H_12_N_2_O_2_ | 204.0898 | 203.0829 | -0.35 | [M-H]^-^ | HMDB0013609 | Level 2 |
| 23 | 3.09 | α-Aspartylphenylalanine | C_13_H_16_N_2_O_5_ | 280.106 | 281.1133 | 0.40 | [M+H]^+^ | HMDB0000706 | Level 2 |
| 24 | 3.53 | N-Phenylacetylglutamine | C_13_H_16_N_2_O_4_ | 264.1112 | 263.1040 | 0.82 | [M-H]^-^ | HMDB0006344 | Level 2 |
| 25 | 3.54 | DL-α-Aminocaprylic acid | C_8_H_17_NO_2_ | 159.1260 | 160.1333 | 0.50 | [M+H]^+^ | HMDB0000991 | Level 2 |
| 26 | 3.61 | Hippuric acid | C_9_H_9_NO_3_ | 179.0584 | 178.0512 | 1.06 | [M-H]^-^ | HMDB0000714 | Level 2 |
| 27 | 3.75 | 3-Methyl-2-Oxovalerate | C_6_H_10_O_3_ | 130.0632 | 129.0559 | 1.48 | [M-H]^-^ | HMDB0000491 | Level 2 |
| 28 | 3.93 | Hexanoylcarnitine | C_13_H_25_NO_4_ | 259.1785 | 260.1858 | 0.57 | [M+H]^+^ | HMDB0000756 | Level 2 |
| 29 | 4.28 | Indole-3-lactic acid | C_11_H_11_NO_3_ | 205.0741 | 204.0669 | 1.09 | [M-H]^-^ | HMDB0000671 | Level 2 |
| 30 | 4.39 | Cinnamoylglycine | C_11_H_11_NO_3_ | 205.0741 | 204.0669 | 1.16 | [M-H]^-^ | HMDB0011621 | Level 2 |
| 31 | 5.31 | Decanoylcarnitine | C_17_H_33_NO_4_ | 315.2412 | 316.2485 | 0.77 | [M+H]^+^ | HMDB0000651 | Level 2 |
| 32 | 5.64 | Testosterone sulfate | C_19_H_28_O_5_S | 368.1660 | 367.1587 | 0.73 | [M-H]^-^ | HMDB0002833 | Level 2 |
| 33 | 6.05 | Glycochenodeoxycholic acid | C_26_H_43_NO_5_ | 449.3146 | 448.3074 | 1.04 | [M-H]^-^ | HMDB0000580 | Level 2 |
| 34 | 6.40 | LPC(14:0) | C_22_H_46_NO_7_P | 467.3014 | 468.3087 | 0.47 | [M+H]^+^ | HMDB0010387 | Level 2 |
| 35 | 6.43 | Sphinganine | C_18_H_39_NO_2_ | 301.2982 | 302.3055 | 0.52 | [M+H]^+^ | HMDB0000269 | Level 2 |
| 36 | 6.49 | Pregnenolone Sulfate | C_21_H_32_O_5_S | 396.1974 | 395.1901 | 0.93 | [M-H]^-^ | HMDB0000774 | Level 2 |
| 37 | 6.64 | LPC(20:5) | C_28_H_48_NO_7_P | 541.3170 | 542.3242 | 0.23 | [M+H]^+^ | HMDB0010397 | Level 2 |
| 38 | 6.65 | LPC(18:3) | C_26_H_48_NO_7_P | 517.3170 | 518.3243 | 0.33 | [M+H]^+^ | HMDB0010388 | Level 2 |
| 39 | 6.80 | LPC(16:1) | C_24_H_48_NO_7_P | 493.3171 | 494.3244 | 0.49 | [M+H]^+^ | HMDB0010383 | Level 2 |
| 40 | 6.90 | Palmitoylcarnitine | C_23_H_45_NO_4_ | 399.3350 | 400.3423 | 0.40 | [M+H]^+^ | HMDB0240774 | Level 2 |
| 41 | 6.93 | LPC(15:0) | C_23_H_48_NO_7_P | 481.3170 | 482.3243 | 0.30 | [M+H]^+^ | HMDB0010379 | Level 2 |
| 42 | 6.94 | LPE(22:6) | C_27_H_44_NO_7_P | 525.2858 | 524.2789 | 0.44 | [M-H]^-^ | HMDB0011526 | Level 2 |
| 43 | 6.95 | LPC(18:2) | C_26_H_50_NO_7_P | 519.3325 | 520.3398 | -0.03 | [M+H]^+^ | HMDB0061700 | Level 2 |
| 44 | 6.98 | LPE(18:2) | C_23_H_44_NO_7_P | 477.2859 | 476.2788 | 0.72 | [M-H]^-^ | HMDB0011507 | Level 2 |
| 45 | 6.98 | LPC(22:6) | C_30_H_50_NO_7_P | 567.3325 | 568.3398 | 0 | [M+H]^+^ | HMDB0010404 | Level 2 |
| 46 | 6.98 | LPE(20:4) | C_25_H_44_NO_7_P | 501.2860 | 500.2789 | 0.89 | [M-H]^-^ | HMDB0011518 | Level 2 |
| 47 | 7.22 | LPC(22:5) | C_30_H_52_NO_7_P | 569.3483 | 570.3556 | 0.31 | [M+H]^+^ | HMDB0010403 | Level 2 |
| 48 | 7.25 | LPC(16:0) | C_24_H_50_NO_7_P | 495.3327 | 496.3400 | 0.35 | [M+H]^+^ | HMDB0010382 | Level 2 |
| 49 | 7.26 | PC(17:1/17:1) | C_42_H_80_NO_8_P | 757.5618 | 758.5691 | -0.44 | [M+H]^+^ | HMDB0286199 | Level 2 |
| 50 | 7.33 | LPC(20:3) | C_28_H_52_NO_7_P | 545.3482 | 546.3555 | 0.17 | [M+H]^+^ | HMDB0010394 | Level 2 |
| 51 | 7.48 | LPE(O-16:1) | C_21_H_44_NO_6_P | 437.2910 | 436.2838 | 0.95 | [M-H]^-^ | HMDB0011152 | Level 2 |
| 52 | 7.48 | LPC(18:1) | C_26_H_52_NO_7_P | 521.3480 | 522.3554 | -0.25 | [M+H]^+^ | HMDB0002815 | Level 2 |
| 53 | 7.67 | LPC(22:4) | C_30_H_54_NO_7_P | 571.3641 | 572.3713 | 0.48 | [M+H]^+^ | HMDB0010401 | Level 2 |
| 54 | 7.80 | LPC(20:2) | C_28_H_54_NO_7_P | 547.3641 | 548.3713 | 0.47 | [M+H]^+^ | HMDB0010392 | Level 2 |
| 55 | 7.86 | LPC(17:0) | C_25_H_52_NO_7_P | 509.3486 | 510.3559 | 0.94 | [M+H]^+^ | HMDB0012108 | Level 2 |
| 56 | 7.98 | LPC(18:0) | C_26_H_54_NO_7_P | 523.3643 | 524.3716 | 0.91 | [M+H]^+^ | HMDB0010384 | Level 2 |
| 57 | 8.12 | PC(O-36:5) | C_44_H_80_NO_7_P | 765.5668 | 766.5742 | -0.54 | [M+H]^+^ | HMDB0013423 | Level 2 |
| 58 | 8.20 | SM(d8:1;2O/34:1) | C_47_H_93_N_2_O_6_P | 812.6763 | 813.6836 | -1.01 | [M+H]^+^ | HMDB0290467 | Level 2 |
| 59 | 8.60 | α-Eleostearic acid | C_18_H_30_O_2_ | 278.2251 | 277.2178 | 1.76 | [M-H]^-^ | HMDB0248208 | Level 2 |
| 60 | 9.04 | Palmitoyl sphingomyelin | C_39_H_79_N_2_O_6_P | 702.5676 | 703.5748 | -0.03 | [M+H]^+^ | HMDB0010169 | Level 2 |
| 61 | 9.69 | 4-Dodecylbenzenesulfonic acid | C_18_H_30_O_3_S | 326.1920 | 325.1848 | 1.43 | [M-H]^-^ | HMDB0059915 | Level 2 |

^*^ is confirmed with standard.
