# Supplementary material for: Integration of metabolomics and machine learning algorithm for discovery of early diagnostic biomarkers of osteoporosis
Source: Metabolomics. 2026 Jul 14;22(4):126. doi: 10.1007/s11306-026-02506-5 (PMC13369700; doi:10.1007/s11306-026-02506-5)
Supplement: Supplementary file 6 — Supplementary Material 6 [file 11306_2026_2506_MOESM6_ESM.docx]

**Table S6. Comparison of diagnostic performance among different models**

| **Model type** | **Covariate adjustment** | **AUC (95% CI)** |
| --- | --- | --- |
| Random Forest (discovery-phase model) | No | 0.985 (0.914–1) |
| LASSO-logistic regression | No | 0.975 (0.936–1) |
| LASSO-logistic regression (final model) | Age and sex adjusted | 0.916 (0.842–0.990) |
