# Supplementary material for: Integration of metabolomics and machine learning algorithm for discovery of early diagnostic biomarkers of osteoporosis
Source: Metabolomics. 2026 Jul 14;22(4):126. doi: 10.1007/s11306-026-02506-5 (PMC13369700; doi:10.1007/s11306-026-02506-5)
Supplement: Supplementary file 7 — Supplementary Material 7 [file 11306_2026_2506_MOESM7_ESM.docx]

**Table S3. Comparison of demographic characteristics and laboratory parameters between the OP and Con groups in the validation cohort [ *x±s*, n (%)].**

| **Index** | **OP group**  **(*n* = 20)** | **Con group**  **(*n* = 42)** | ***t/χ²*** | ***p*** |
| --- | --- | --- | --- | --- |
| Gender (male/female) | 7/13 | 14/28 | <0.001 | 1.000 |
| Age (year) | 65.20 ± 11.05 | 61.05 ± 5.93 | –1.576 | 0.128 |
| Height (m) | 1.62 ± 0.04 | 1.67 ± 0.08 | 2.935 | 0.005 |
| Weight (kg) | 60.02 ± 8.29 | 60.95 ± 11.15 | 0.328 | 0.744 |
| BMI (kg/m²) | 22.84 ± 2.95 | 22.16 ± 5.05 | –0.667 | 0.508 |
| ALT (U/L) | 15.23 ± 6.08 | 18.56 ± 7.96 | 1.653 | 0.104 |
| AST (U/L) | 16.67 ± 4.45 | 18.01 ± 3.88 | 1.207 | 0.232 |
| HDL (mmol/L) | 1.18 ± 0.29 | 1.43 ± 0.24 | 3.529 | <0.001 |
| LDL (mmol/L) | 3.02 ± 1.06 | 2.50 ± 0.53 | –2.089 | 0.048 |
| Lumbar T-score | –2.69 ± 0.81 | 0.85 ± 0.54 | 17.695 | <0.001 |
| Hip T-score | –2.66 ± 0.73 | –0.04 ± 0.55 | 15.798 | <0.001 |
| Vitamin D (nmol/L) | 16.08 ± 5.71 | 14.50 ± 2.62 | –1.183 | 0.249 |
| P (mmol/L) | 1.25 ± 0.10 | 1.19 ± 0.13 | –1.807 | 0.076 |
| Ca (mmol/L) | 2.32 ± 0.18 | 2.34 ± 0.15 | 0.380 | 0.706 |
