# Supplementary material for: Integration of metabolomics and machine learning algorithm for discovery of early diagnostic biomarkers of osteoporosis
Source: Metabolomics. 2026 Jul 14;22(4):126. doi: 10.1007/s11306-026-02506-5 (PMC13369700; doi:10.1007/s11306-026-02506-5)
Supplement: Supplementary file 8 — Supplementary Material 8 [file 11306_2026_2506_MOESM8_ESM.docx]

**Table S2.** **Statistical parameters of the 61 differential metabolites after adjustment for age and sex**

| **Metabolites** | **VIP** | ***p*** | **FC** | **FDR** |
| --- | --- | --- | --- | --- |
| L-Lysine | 1.31 | 1.54E-58 | 1.48 | 2.68E-58 |
| Pipecolic acid | 1.28 | 9.73E-53 | 1.37 | 1.48E-52 |
| Valine | 1.43 | 3.89E-136 | 9.68 | 2.64E-135 |
| Glycerophosphocholine | 1.38 | 2.02E-91 | 3.30 | 5.35E-91 |
| Proline | 1.33 | 6.17E-64 | 2.17 | 1.18E-63 |
| Choline | 1.42 | 1.57E-126 | 2.91 | 8.71E-126 |
| DL-Carnitine | 1.42 | 5.23E-136 | 4.28 | 3.19E-135 |
| Hypoxanthine | 1.42 | 6.48E-125 | 3.21 | 3.04E-124 |
| Methionine | 1.40 | 5.11E-103 | 2.41 | 1.56E-102 |
| Prolylleucine | 1.36 | 3.49E-77 | 1.94 | 7.87E-77 |
| L-Pyroglutamic acid | 1.43 | 2.22E-146 | 3.64 | 3.38E-145 |
| Uracil | 1.33 | 1.3E-66 | 1.71 | 2.64E-66 |
| Uric acid | 1.02 | 5.92E-58 | 2.74 | 1E-57 |
| Citric acid | 1.26 | 2.24E-53 | 1.50 | 3.51E-53 |
| 3-Hydroxybutyric acid | 1.11 | 1.67E-32 | 1.38 | 1.89E-32 |
| L-Tyrosine | 1.16 | 1.05E-36 | 1.22 | 1.29E-36 |
| Propionylcarnitine | 1.12 | 7.69E-30 | 1.47 | 8.53E-30 |
| L-Norleucine | 1.43 | 2.45E-136 | 4.79 | 1.87E-135 |
| L-Phenylalanine | 1.07 | 2.51E-25 | 1.45 | 2.6E-25 |
| 6-Methylquinoline | 1.22 | 5.79E-41 | 1.23 | 7.35E-41 |
| trans-3-Indoleacrylic acid | 1.42 | 7.74E-123 | 2.64 | 3.37E-122 |
| DL-Tryptophan | 1.43 | 3.5E-144 | 2.09 | 3.05E-143 |
| α-Aspartylphenylalanine | 1.37 | 3.94E-85 | 1.98 | 1E-84 |
| N-Phenylacetylglutamine | 1.21 | 8.94E-48 | 1.87 | 1.21E-47 |
| DL-α-Aminocaprylic acid | 1.15 | 1.35E-34 | 1.28 | 1.55E-34 |
| Hippuric acid | 1.26 | 4.12E-51 | 2.02 | 6.13E-51 |
| 3-Methyl-2-Oxovalerate | 1.43 | 5.18E-155 | 7.99 | 1.05E-153 |
| Hexanoylcarnitine | 1.08 | 5.07E-25 | 1.31 | 5.15E-25 |
| Indole-3-lactic acid | 1.27 | 4.23E-51 | 1.46 | 6.15E-51 |
| Cinnamoylglycine | 1.31 | 1.54E-58 | 1.48 | 2.68E-58 |
| Decanoylcarnitine | 1.29 | 2.01E-57 | 1.36 | 3.32E-57 |
| Testosterone sulfate | 1.25 | 1.45E-46 | 2.13 | 1.92E-46 |
| Glycochenodeoxycholic acid | 1.42 | 2.34E-126 | 4.64 | 1.19E-125 |
| LPC(14:0) | 1.32 | 5.31E-67 | 2.35 | 1.12E-66 |
| Sphinganine | 1.29 | 8.13E-61 | 1.72 | 1.46E-60 |
| Pregnenolone Sulfate | 1.03 | 4.09E-26 | 1.29 | 4.38E-26 |
| LPC(20:5) | 1.12 | 9.01E-26 | 1.48 | 9.47E-26 |
| LPC(18:3) | 1.41 | 1.12E-111 | 3.40 | 4.02E-111 |
| LPC(16:1) | 1.40 | 5.82E-108 | 3.68 | 1.97E-107 |
| Palmitoylcarnitine | 1.39 | 6.99E-94 | 5.30 | 1.94E-93 |
| LPC(15:0) | 1.04 | 1.03E-22 | 1.38 | 1.03E-22 |
| LPE(22:6) | 1.15 | 5.1E-36 | 1.67 | 5.98E-36 |
| LPC(18:2) | 1.36 | 8.88E-82 | 2.10 | 2.17E-81 |
| LPE(18:2) | 1.44 | 9.98E-208 | 1.33 | 6.09E-206 |
| LPC(22:6) | 1.36 | 8.34E-80 | 3.23 | 1.96E-79 |
| LPE(20:4) | 1.42 | 5.82E-121 | 1.99 | 2.37E-120 |
| LPC(22:5) | 1.40 | 3.3E-104 | 3.52 | 1.06E-103 |
| LPC(16:0) | 1.41 | 8.14E-116 | 7.60 | 3.1E-115 |
| PC(17:1/17:1) | 1.43 | 4.28E-145 | 1.50 | 5.16E-144 |
| LPC(20:3) | 1.20 | 5.35E-37 | 6.14 | 6.66E-37 |
| LPE(O-16:1) | 1.44 | 6.42E-171 | 1.22 | 1.96E-169 |
| LPC(18:1) | 1.32 | 1.45E-61 | 3.51 | 2.68E-61 |
| LPC(22:4) | 1.43 | 5.08E-145 | 4.05 | 5.16E-144 |
| LPC(20:2) | 1.25 | 2.05E-48 | 2.13 | 2.91E-48 |
| LPC(17:0) | 1.38 | 1.65E-94 | 5.45 | 4.79E-94 |
| LPC(18:0) | 1.33 | 4.41E-65 | 10.40 | 8.68E-65 |
| PC(O-36:5) | 1.25 | 8.71E-48 | 2.58 | 1.21E-47 |
| SM(d8:1;2O/34:1) | 1.21 | 1.16E-41 | 4.76 | 1.5E-41 |
