# Supplementary material for: Integration of metabolomics and machine learning algorithm for discovery of early diagnostic biomarkers of osteoporosis
Source: Metabolomics. 2026 Jul 14;22(4):126. doi: 10.1007/s11306-026-02506-5 (PMC13369700; doi:10.1007/s11306-026-02506-5)
Supplement: Supplementary file 9 — Supplementary Material 9 [file 11306_2026_2506_MOESM9_ESM.docx]

**Table S5. Random forest model hyperparameters and performance metrics** **after adjustment**

|  | **Parameter / Metric** | **Value** |
| --- | --- | --- |
| Model Configuration | Number of trees (*ntree*) | 500 |
|  | Variables per split (*mtry*) | 3 |
|  | Cross-validation strategy | 10-fold CV |
|  | Out-of-bag (OOB) error | 6.2% |
| Performance Metrics | AUC (95% CI) | 0.916 |
|  | Sensitivity (95% CI) | 75% |
|  | Specificity (95% CI) | 88.1% |
|  | Accuracy | 82.9% |
