# Supplementary material for: Integration of metabolomics and machine learning algorithm for discovery of early diagnostic biomarkers of osteoporosis
Source: Metabolomics. 2026 Jul 14;22(4):126. doi: 10.1007/s11306-026-02506-5 (PMC13369700; doi:10.1007/s11306-026-02506-5)
Supplement: Supplementary file 10 — Supplementary Material 10 [file 11306_2026_2506_MOESM10_ESM.docx]

**Table S4. Statistical parameters of the 4 lipids before and after adjustment for age and sex**

| **Lipids** | **Before adjustment** | | | **After adjustment** | | |
| --- | --- | --- | --- | --- | --- | --- |
|  | ***p*** | **FC** | **VIP** | ***p*** | **FC** | **VIP** |
| LPA(16:0) | 5.12E-59 | 1.59 | 1.84 | 8.03E-25 | 1.31 | 1.38 |
| LPI(16:0) | 1.80E-41 | 1.50 | 1.85 | 1.87E-19 | 1.25 | 1.38 |
| LPI(18:0) | 1.80E-21 | 1.57 | 1.45 | 4.01E-09 | 1.29 | 1.38 |
| LPI(20:0) | 1.68E-21 | 1.91 | 1.51 | 1.43E-09 | 1.97 | 1.37 |
